# Inhibition of EV71 by curcumin in intestinal epithelial cells

**DOI:** 10.1371/journal.pone.0191617

**Published:** 2018-01-25

**Authors:** Hsing-I Huang, Chi-Chong Chio, Jhao-Yin Lin

**Affiliations:** 1 Research Center for Emerging Viral Infections, College of Medicine, Chang Gung University, Kwei-Shan, Tao-Yuan, Taiwan; 2 Department of Medical Biotechnology and Laboratory Science, College of Medicine, Chang Gung University, Kwei-Shan, Tao-Yuan, Taiwan; 3 Graduate Institute of Biomedical Sciences, College of Medicine, Chang Gung University, Kwei-Shan, Tao-Yuan, Taiwan; 4 Department of Pediatrics, Chang Gung Memorial Hospital, Linkou, Taiwan; University of Minnesota College of Veterinary Medicine, UNITED STATES

## Abstract

EV71 is a positive-sense single-stranded RNA virus that belongs to the *Picornaviridae* family. EV71 infection may cause various symptoms ranging from hand-foot-and-mouth disease to neurological pathological conditions such as aseptic meningitis, ataxia, and acute transverse myelitis. There is currently no effective treatment or vaccine available. Various compounds have been examined for their ability to restrict EV71 replication. However, most experiments have been performed in rhabdomyosarcoma or Vero cells. Since the gastrointestinal tract is the entry site for this pathogen, we anticipated that orally ingested agents may exert beneficial effects by decreasing virus replication in intestinal epithelial cells. In this study, curcumin (diferuloylmethane, C_21_H_20_O_6_), an active ingredient of turmeric (*Curcuma longa* Linn) with anti-cancer properties, was investigated for its anti-enterovirus activity. We demonstrate that curcumin treatment inhibits viral translation and increases host cell viability. Curcumin does not exert its anti-EV71 effects by modulating virus attachment or virus internal ribosome entry site (IRES) activity. Furthermore, curcumin-mediated regulation of mitogen-activated protein kinase (MAPK) signaling pathways is not involved. We found that protein kinase C delta (PKCδ) plays a role in virus translation in EV71-infected intestinal epithelial cells and that curcumin treatment decreases the phosphorylation of this enzyme. In addition, we show evidence that curcumin also limits viral translation in differentiated human intestinal epithelial cells. In summary, our data demonstrate the anti-EV71 properties of curcumin, suggesting that ingestion of this phytochemical may protect against enteroviral infections.

## Introduction

EV71 is a positive-sense single-stranded RNA virus of the family *Picornaviridae*. Other notable members of this family include poliovirus, coxsackievirus, EV-D68, and rhinovirus. These pathogens are known for their highly contagious properties, susceptibility to mutation, and ability to cause serious diseases. EV71 outbreaks have occurred in the Asia-Pacific region over the past two decades [1, 2]. The pathological conditions caused by EV71 infection include hand-foot-and-mouth disease, herpangina, and neurological complications. EV71-induced neurological diseases may result in pulmonary edema, which is the major cause of death in EV71 patients [3]. There are currently no effective therapeutic agents or available vaccines for EV71 infection. Therefore, it is important to identify anti-EV71 agents to control the spread of this virus.

Enteroviruses replicate in the gastrointestinal tract and are then disseminated to other organs/tissues. For other gut pathogens such as rotavirus and norovirus, compounds that can decrease intestinal viral titers have been shown to exert therapeutic effects in animal experiments [4]. A recent study showed that the ingestion of rice bran protects against rotavirus-induced diarrhea in pigs [5]. Therefore, phytochemical ingestion has the potential to combat enteric pathogens. However, oral compounds have not been examined for their ability to treat enterovirus infections.

Phytochemical ingestion has proven to be effective in the prevention of cancer [6]. However, whether dietary phytochemicals can be applied to treat viral diseases is not clear. Curcumin is a natural phytochemical extracted from the rhizome of the plant *Curcuma longa* L. [7]. In addition to its use as a dye, curcumin has long been used to promote wound healing and treat inflammatory conditions. Curcumin is safe for human consumption, even at high doses, and few side effects have been reported in animal studies and human trials. Thus, curcumin is ideal for use in medical applications. Several clinical trials have been performed to test the effectiveness of curcumin in cancer prevention, with some showing encouraging results [8]. Accumulating evidence suggests that curcumin also exerts antiviral activities, and HCV and HBV replication is down-regulated by curcumin treatment [9,10]. A curcumin-containing diet has been shown to effectively inhibit diethylnitrosamine-induced hepatocarcinogenesis and acute small intestinal inflammation in animals [11,12]. However, whether curcumin treatment can exert antiviral activity in intestinal epithelial cells has not been investigated. In this study, we examined the anti-EV71 activity and associated mechanisms of curcumin in intestinal epithelial cells. Our results show that the ability of curcumin to modulate PKCδ activation may contribute to its anti-EV71 activity.

## Methods

### Cells and virus

HT29 cells were a kind gift from Dr. Jason C. Huang (National Yang Ming University, Taipei). These cells were expanded in RPMI-1640 medium supplemented with 10% fetal bovine serum (FBS) (all from Thermo-Fisher Scientific, MA, USA). C2BBe1 cells were obtained from Dr. Chiu Cheng-Hsun (Department of Pediatrics, Chang-Gung Memorial Hospital, Kwei-Shan Tao-Yuan) and expanded in Dulbecco's modified Eagle medium (DMEM) supplemented with 10% FBS and 0.01 mg/ml human transferrin (all from Thermo-Fisher Scientific, MA, USA). Human rhabdomyosarcoma (RD) cells were obtained from Dr. Shih Shin-Ru and maintained in DMEM containing 10% FBS, 1% non-essential amino acids, 1% L-glutamine, and 1% penicillin/streptomycin (all from Thermo-Fisher Scientific, MA, USA). All cells were incubated in a 37°C incubator equilibrated with 5% CO_2_. The clinically isolated EV71 strain 4643 (EV71/Tainan/4643/98) was amplified in RD cells, and the titer was quantified by plaque forming assays.

### Viral infection

Cells were seeded on 12-well plates at a density of 2x 10^5^ cells per well for 48 hours before infection. The wells were rinsed twice with phosphate-buffered saline (PBS), and virus was then added to the cells at a specified multiplicity of infection (MOI) in serum-free medium. After adsorption at 37°C for 1 hour, the virus-containing medium was removed and then washed by PBS twice. Medium containing 2% serum was then added to the cells for subsequent incubation. For infection assays involving curcumin treatment, the cells were pretreated with curcumin for one hour, and the adsorption and infection steps were carried out in the presence of the tested reagent.

### Differentiation of C2BBe1 intestinal epithelial cells

C2BBe1 cells were seeded on plates at a density of 5 x 10^5^ cells/cm^2^ and maintained in a mixture of intestinal epithelium differentiation medium (Corning, MA, USA) and C2BBe1 culture medium (1:1). After 24 hours, the medium was changed to full intestinal epithelium differentiation medium with 1% ITS-A Supplement (Thermo-Fisher Scientific, MA, USA), and the cells were incubated for 48 hours.

### Reagents

Curcumin, apigenin, dextran sulfate and rottlerin (purchased from Sigma-Aldrich, MO, USA) compounds were obtained, and 10 mM stock solutions were prepared in DMSO. The final concentration of rottlerin used was 5 μM. PR66 was obtained from Dr. Jim-Tong Horng (Chang Gung University, Taoyuan).

### Immunofluorescence staining

For immunofluorescence staining, cells were fixed with ice-cold 4% paraformaldehyde at 4°C for 10 min. After being washed with 1× PBS three times, the cells were permeabilized with 0.5% Triton X-100 for 5 min. The cells were then washed with 1× PBS three times and blocked with PBS containing 2% FBS (Thermo-Fisher Scientific, MA, USA) at room temperature for 30 min. A mouse anti-EV71 3D antibody (1:800, a kind gift from Professor Shin-Ru Shih, Chang Gung University) was added to the cells and incubated overnight at 4°C. After three washes for 5 min each, an anti-dylight 594 mouse IgG secondary antibody (1:1000, Thermo-Fisher Scientific, MA, USA) was added to bind to the primary Ab at room temperature for one hour. Unbound antibody was then removed by washing three times for 5 min each. DAPI (4',6-diamidino-2-phenylindole) (Sigma-Aldrich, MO, USA) was then applied counterstain the cell nuclei. The results were observed using a fluorescence microscope (Olympus IX71).

### Growth curve of infected cells

HT-29 cells were seeded in 12 well plates and infected with EV71 at the MOI of 1 in the absence or presence of curcumin (10 μM). The numbers of cells were counted by trypan blue exclusion assay at 12, 24, 36 and 48 hours p.i. Per condition was performed in triplicate.

### Compound binding assay

Virus was mixed with the tested compound for one hour on ice and then used to infect HT29 cells for 12 hours. Total cell lysates were obtained and subjected to Western blot analysis.

### Virucidal activity assay

Virus was mixed with curcumin at 0, 5, 10 and 20 μM in room temperature for 1hr and then infected HT-29. After adsorption, virus and compound were removed and cells were washed with PBS. Cells were incubated in 2% FBS medium at 37°C, 5% CO_2_ and total cell lysates were extracted at 12 hr p.i. and analyzed the expression of viral protein by Western blot.

### Virus attachment assay

Cells were infected with EV71 at the MOI of 1 in the presence or absence of curcumin, dextran sulfate or PR66 on ice for one hour. The compound was the removed by aspiration and the cells were washed by PBS. The cells were incubated in 2% FBS medium for 48 hr and determined the cellular viability by MTT assay.

### Cytotoxicity assay

To determine the cytotoxicity of curcumin, HT29 cells were seeded in 48-well plates and incubated with different concentrations of tested reagents in triplicate. After 48 hours of incubation, the cells were harvested and stained with trypan blue solution. The cells were counted using a hemocytometer. MTT assay was also performed to assess the cell viability. MTT [3-(4,5-dimethylthiazol-2-yl)-2,5-diphenyltetrazolium bromide] (Sigma-Aldrich, MO, USA) was obtained and prepared in PBS for the stock solution (5mg/ml). Culture medium was removed and then cells were washed with PBS. MTT stock solution was diluted 5x using PBS or serum free medium and added to the cells and incubated at 37°C, 5% CO_2_ for 2 hr. The MTT solution was aspirated and 0.04N HCl in isopropanol was added to cells for dissolved the formazan crystals. The color intensity was measured at the 570 nm using an ELISA reader.

### Time-of-addition assay

HT29 cells cultured in 12-well plates were infected with EV71 at the MOI of 1. Curcumin was added at a final concentration of 10 μM at the different time points. Total cell lysates were collected, and viral protein expression levels were detected by Western blot analysis.

### Western blot

Cultured cells were washed with PBS, and total cell lysates were collected by incubating the cells for 30 min on ice in ice-cold protein lysis buffer (1% NP-40, 50 mM Tris, and 150 mM NaCl) supplemented with a protease inhibitor cocktail. After centrifugation at 13,000 rpm for 10 min at 4°C, the supernatant and pellet fractions were collected. Protein concentrations were measured using the Bradford method (Bio-Rad Laboratories, CA, USA), and 30 μg of protein was separated by 10% SDS-polyacrylamide gel electrophoresis and then transferred onto a polyvinylidene fluoride membrane (PVDF) (GE, MA, USA). The protein-containing membrane was blocked with 5% skim milk in Tris-buffered saline Tween-20 buffer (TBST, 20 mM Tris-HCl, pH 7.4, 150 mM NaCl, and 0.1% Tween 20) at room temperature. The membrane was then incubated with antibodies against EV71 3D (1:10,000, a kind gift from Professor Shin-Ru Shih, Chang Gung University), EV71 VP1 (1:2000, Merck Millipore, MA, USA), total Erk (1:1000), P-Erk (1:1000), total p38 (1:1000), P-p38 (1:1000), total PKCδ (1:2000), P-PKCδ (Tyr311) (1:1000), p-JNK (1:1000), p-c-Jun (1:1000), E-cadherin (1:1000), Villin (1:1000) (all from Cell Signaling, CA, USA), SOX9 (1:2000, Merck Millipore, MA, USA), CDX2 (1:1000, GeneTex, Taiwan) or β-actin (1:20,000, Sigma-Aldrich, MO, USA) and probed with anti-rabbit or anti-mouse secondary antibody conjugated horseradish peroxidase (1:5000, Jackson ImmunoResearch Laboratories, Pennsylvania, USA). The target proteins were visualized with a chemiluminescence reagent (PerkinElmer, MA, USA).

### RNA extraction and real-time PCR

Total RNA was isolated with TRIzol reagent (Thermo-Fisher Scientific, MA, USA), and 1μg of total RNA was used to synthesize cDNA using a RevertAid First Strand cDNA Synthesis Kit (Thermo-Fisher Scientific, MA, USA) according to the manufacturer's instructions. The primers used in the assay were kind gifts from Professor Shin-Ru, Shih (Chang Gung University, Taoyuan). qPCR analysis was performed with 1 μl of each cDNA sample, 5 μM forward and reverse primers, and SYBR green (KAPA Biosystems, MA, USA) was used to quantify gene expression. qPCR assays were carried out in 384-well plates and analyzed by a Roche Lightcycler 480 instrument. Each sample was assayed in triplicate, and β-actin was used as a reference gene. We analyzed the relative quantification of each gene using the 2^-ΔΔCT^ method.

### Plaque assay

RD cells were seeded in 6-well plates at a density of 5.5 x 10^5^ cells per well and incubated at 37°C in 5% CO_2_ for 20 to 24 hours. The cells were then washed once with PBS and infected with 500 μl of serially diluted virus suspensions in serum-free medium. After 1 hour of adsorption at 37°C in 5% CO_2_, the culture medium was removed, and the cells were washed twice with PBS to remove unbound viruses. Afterward, 3 ml of DMEM containing 2% FBS and 0.3% agarose gel was added to each well. After 4 days, the cells were fixed with 10% formaldehyde for more than 1 hour and subsequently stained with 0.5% crystal violet solution. The virus titer was expressed as plaque forming units (pfu) per milliliter.

### *In vitro* transcription

The pGL3-EV71 5′UTR-FLuc plasmid (a kind gift from Dr. Shin-Ru Shih, Chang Gung University) was digested with XbaI (New England Biolabs, MA, USA) to form a linear template for *in vitro* transcription. EV71 5′UTR-FLuc RNA was synthesized by using a MEGA script T7 Kit (Ambion, CA, USA). According to the manufacturer's instructions, the MEGA script transcription reaction mixture was prepared, containing 75 mM ATP, 75 mM UTP, 75 mM CTP, 75 mM GTP (2 μl of each nucleotide), 2 μl of 10× reaction buffer, 1 μg of linearized DNA template, 2 μl of enzyme mix and nuclease-free water to bring the volume to 20 μl. The mixture was incubated at 37°C for 4 hours, and 1μl of TURBO DNase (2 U/l) (Thermo-Fisher, MA, USA) was added for an additional incubation at 37°C for 15 min. The RNA product was purified by using an RNeasy Protect Mini Kit (Qiagen, NW, Germany) and stored at -80°C.

### Preparation of HT-29 translation cell lysate

HT29 cells were trypsinized and centrifuged at 1000 rpm for 5 min at 4°C. The cell pellet was weighed and resuspended in 1.5 volume of hypotonic lysis buffer {10 mM HEPES-KOH [pH 7.6], 10 mM potassium acetate [CH_3_CO_2_K], 2.5 mM magnesium acetate [Mg(CH_3_CO_2_)_2_], 2 mM DTT and EDTA free-protease inhibitor} (all from Sigma-Aldrich, MO, USA) on ice for 45 min. The cells were homogenized by syringe (1 ml volume, 27G, 3/4inch needle), and lysis of most of the cells (> 90%) was confirmed by trypan blue staining. The cell lysate was centrifuged at 10,400 × g for 20 min at 4°C, and the supernatant was stored at -80°C.

### *In vitro* translation assay

*In vitro* translation assays for detecting EV71 IRES activity were performed by mixing 0.5 μg EV71 5’UTR-FLuc RNA, 80 μg HT-29 cell lysate, tested compound (5, 10 and 20 μM curcumin, 30 and 100 μM apigenin), 20% rabbit reticulocyte lysates (RRL) (Promega, WI, USA), 0.25 μl Methionine, 0.25 μl leucine and nuclease-free water to 25 μl and incubation at 30°C for 1.5 hr. Finally, 10 μl of each sample was mixed with 50 μl of luciferase reagent from the Luciferase Assay System (Promega, WI, USA) to measure the firefly luciferase activity.

### Statistical analysis

Measurement data were expressed as the mean ± standard deviation. Comparisons among different groups were analyzed by Student’s two-tailed t-test. Statistical significant was defined as *, *p* <0.05, **, *p* <0.01, and ***, *p* <0.001.

## Results

### Human intestinal epithelial HT-29 cells are permissive to EV71 infection

**Fig 1:**
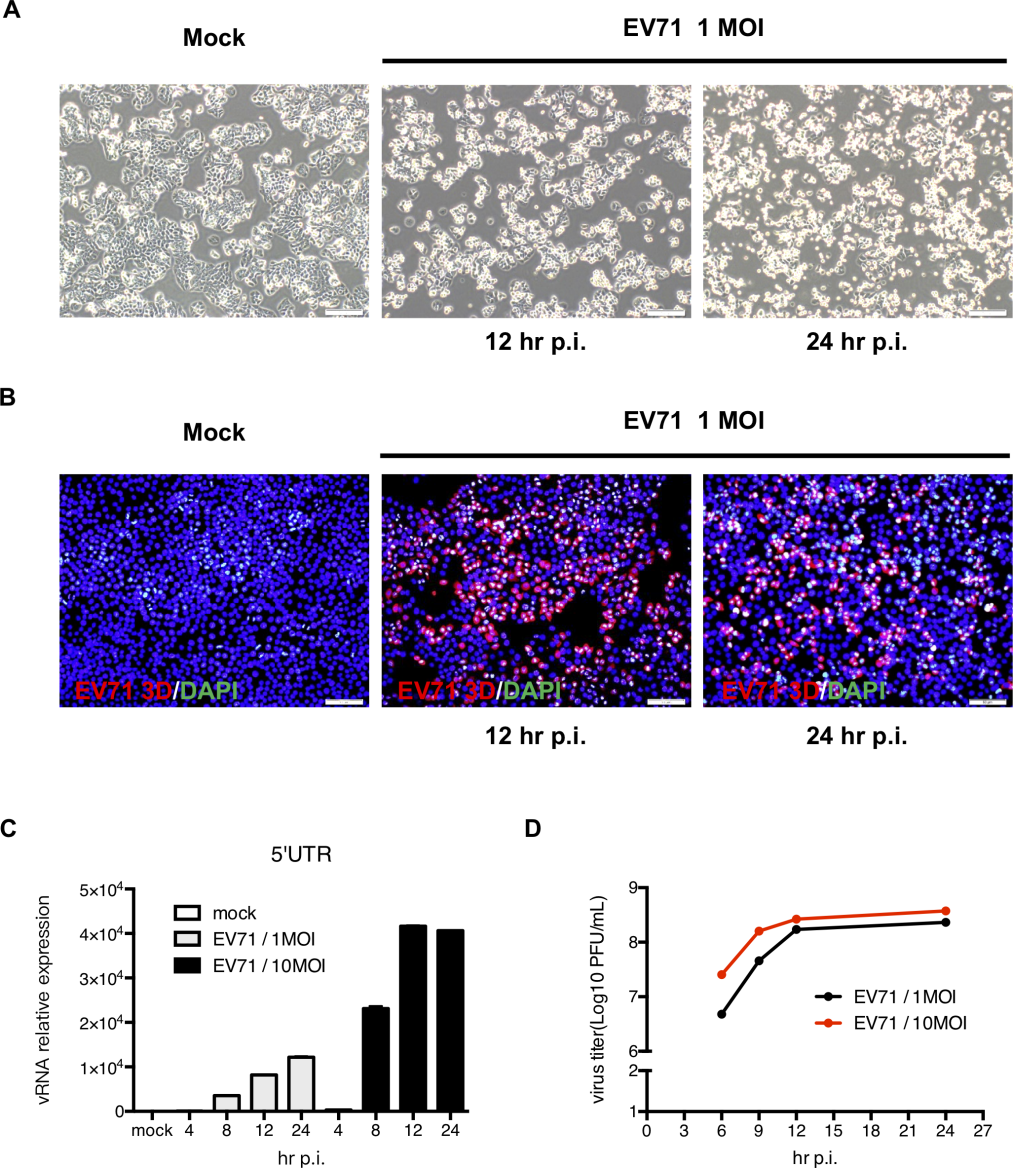
HT-29 cells are permissive to EV71 infection. (A) HT29 cells were seeded on culture plates and infected with EV71 at the MOI of 1. Cell morphology was observed using an inverted microscope (magnification = 200x). (B) To confirm infection, the cells were fixed and reacted with a primary anti-EV71 3D antibody. A PE-conjugated anti-mouse IgG antibody was then applied. DAPI was used to stain the cell nuclei (magnification = 200x). (C) Total RNA was extracted from mock-infected and EV71-infected cells, and RT-qPCR was performed to detect the quantity of viral RNA. (D) Total cell lysates were harvested to detect the viral titers using a plaque assay.

To examine the susceptibility of gut epithelial cells to EV71 infection, HT29 human intestinal epithelial cells were infected with EV71 at an MOI of 1 (Fig 1A). After 12 hours of infection, the cells gradually detached from the culture plates, cytopathic effects were evident, and cytoplasmic shrinkage could be observed in most cells. To examine whether these cells were infected with EV71, the expression of the EV71 viral protein 3D was detected by immunofluorescence staining. As shown in Fig 1B, EV71 3D could be detected in most cells after 12 hours of infection. In addition to viral protein expression, active caspase 3 expression was also examined (S1 Fig). Active caspase 3 and cell nuclei shrinkage could be observed in EV71-infected HT29 cells. Therefore, EV71 infection induces apoptosis in intestinal epithelial cells. Furthermore, RNA samples were collected at different time points, and the expression levels of EV71 RNA were quantified by RT-qPCR. The expression of EV71 5’UTR RNA increased proportionally with infection time (Fig 1C). The production of virus progeny peaked at 12 hours post-infection (p.i.) in HT29 cells (Fig 1D). These results revealed that EV71 actively replicates in intestinal cells, in accordance with previous observations [13].

### Curcumin treatment decreases cell death and virus replication in intestinal epithelial cells

**Fig 2:**
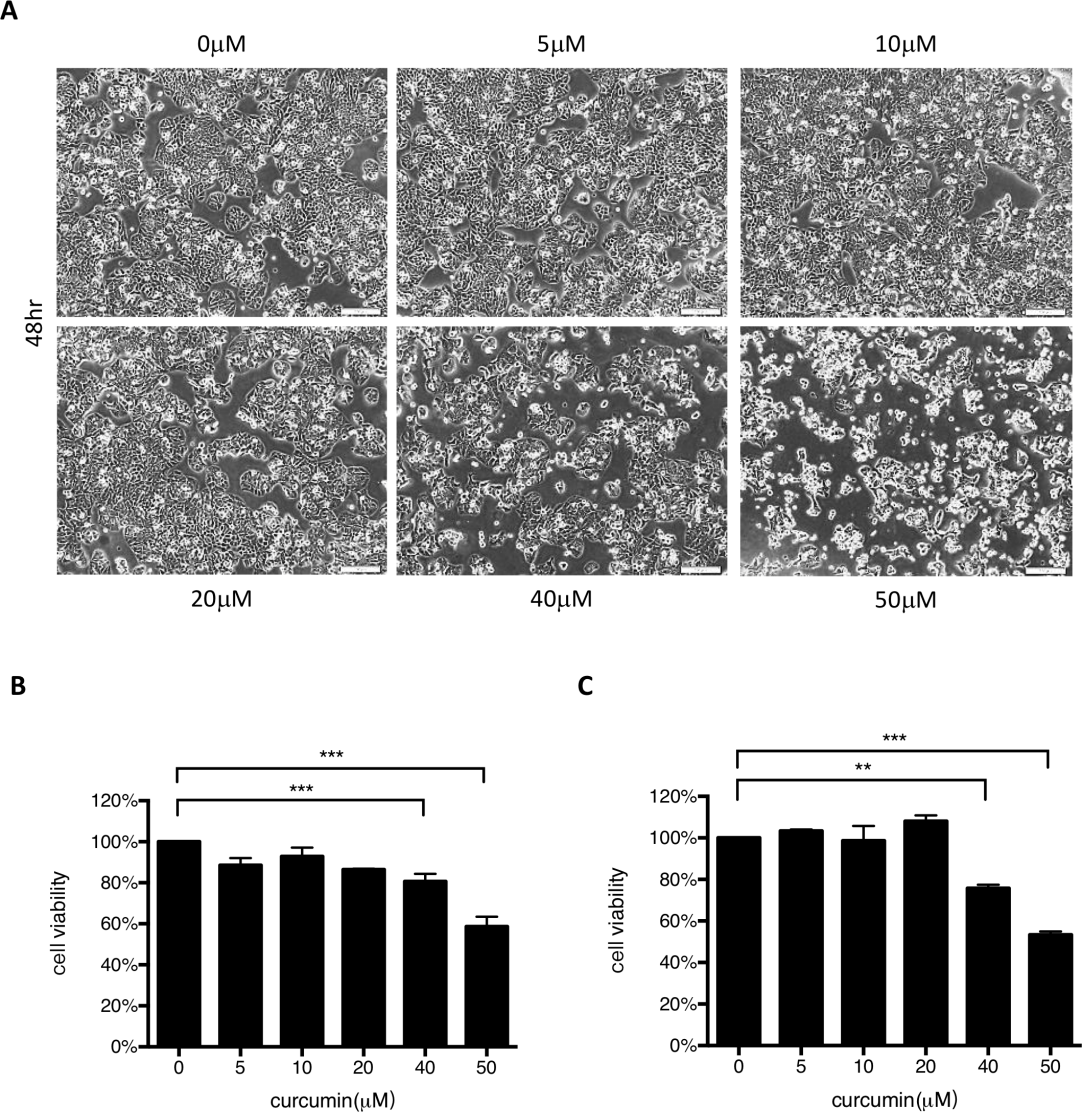
Curcumin cytotoxicity assay. **(A)** To detect the cytotoxicity of curcumin in host cells, HT29 cells were seeded and treated with various concentrations of curcumin for 2 days. Cell morphology was observed and recorded (magnification = 200x). (B) Trypan blue exclusion and (C) MTT assays were performed to determine the viability of treated cells.

A cytotoxicity assay was performed to examine which concentrations of curcumin caused the significant HT29 cell death. Cells were seeded on culture plates and treated with curcumin at different concentrations for 48 hours. Cell morphology was observed under a light microscope. Cells treated with >40 μM curcumin detached from the culture plate (Fig 2A). An MTT assay and trypan blue exclusion assay were performed to determine cell viability. In both assays, significant cell death was observed in cells treated with higher concentrations of curcumin (Fig 2B and 2C).

**Fig 3:**
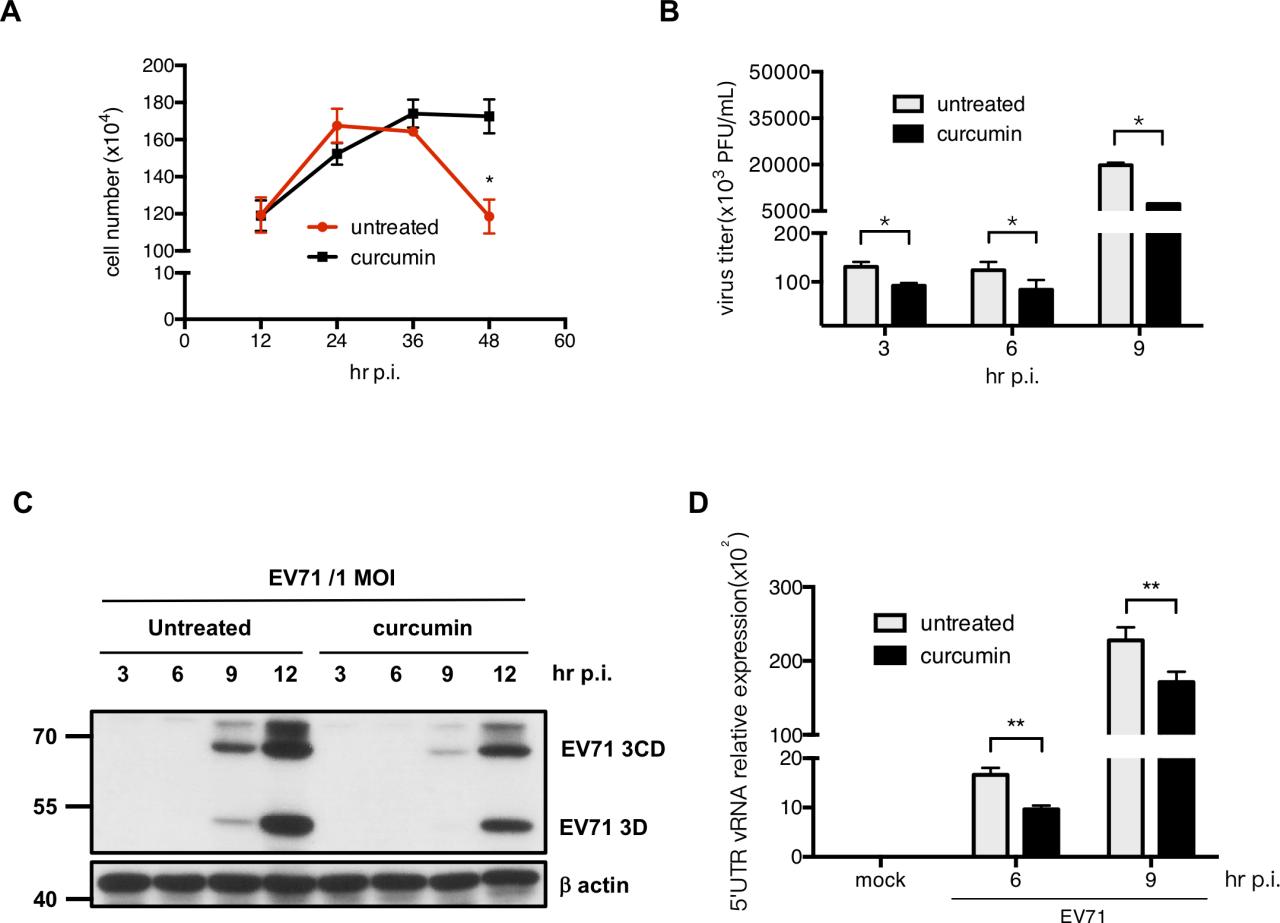
Curcumin treatment increases the survival of host cells and suppresses EV71 replication. (A) Cells were seeded in culture plates and treated with 10 μM curcumin. After one hour, the medium was removed, and the cells were infected with EV71 at the MOI of 1 in curcumin-containing serum-free medium. After adsorption, the medium was removed, and medium containing 2% serum and 10 μM curcumin was added. The growth curves of infected cells were counted using a trypan blue exclusion assay. (B) Cell lysates were collected from cell samples, and a plaque assay was performed to determine the virus titers. (C) Cells were harvested at different time points, and total protein was extracted to determine the expression of EV71 3D protein by Western blot. The expression of β-actin was used as an internal control. (D) RT-qPCR analysis was performed to detect the amounts of viral RNA.

To examine the ability of curcumin to protect cells from EV71-induced cell death, we analyzed the numbers of cells infected with EV71 in the absence or presence of curcumin. Upon EV71 infection, the numbers of curcumin-treated cells were higher than that of untreated cells (Fig 3A). This result is in accordance with the previous observation that curcumin is able to suppress CVB3-induced CPE and apoptosis [14]. To investigate the antiviral effects of curcumin, HT29 cells were treated with curcumin and then infected with EV71. The virus titer was decreased in curcumin-treated cells compared with untreated cells (Fig 3B). PR66, an imidazolidinone derivative, was used as a control in this experiment (S2 Fig) [15]. Interestingly, we found that in addition to EV71, curcumin also suppressed the replication of CVB3 and EV-D68 viruses in intestinal epithelial cells (S3 Fig). To determine whether curcumin exerts effects on viral protein expression, HT29 cells treated with 10 μM curcumin were infected with EV71 at an MOI of 1, and protein samples were collected at 3, 6, 9, and 12 hours p.i. Western blot analysis was then performed to determine the expression levels of the EV71 viral protein 3D. Curcumin treatment exerted inhibitory effects on viral protein expression (Fig 3C). RT-qPCR was also performed and showed that curcumin suppressed the replication of viral genomic RNA during the early stage of infection (Fig 3D).

### Curcumin acts at early stages of the virus life cycle

**Fig 4:**
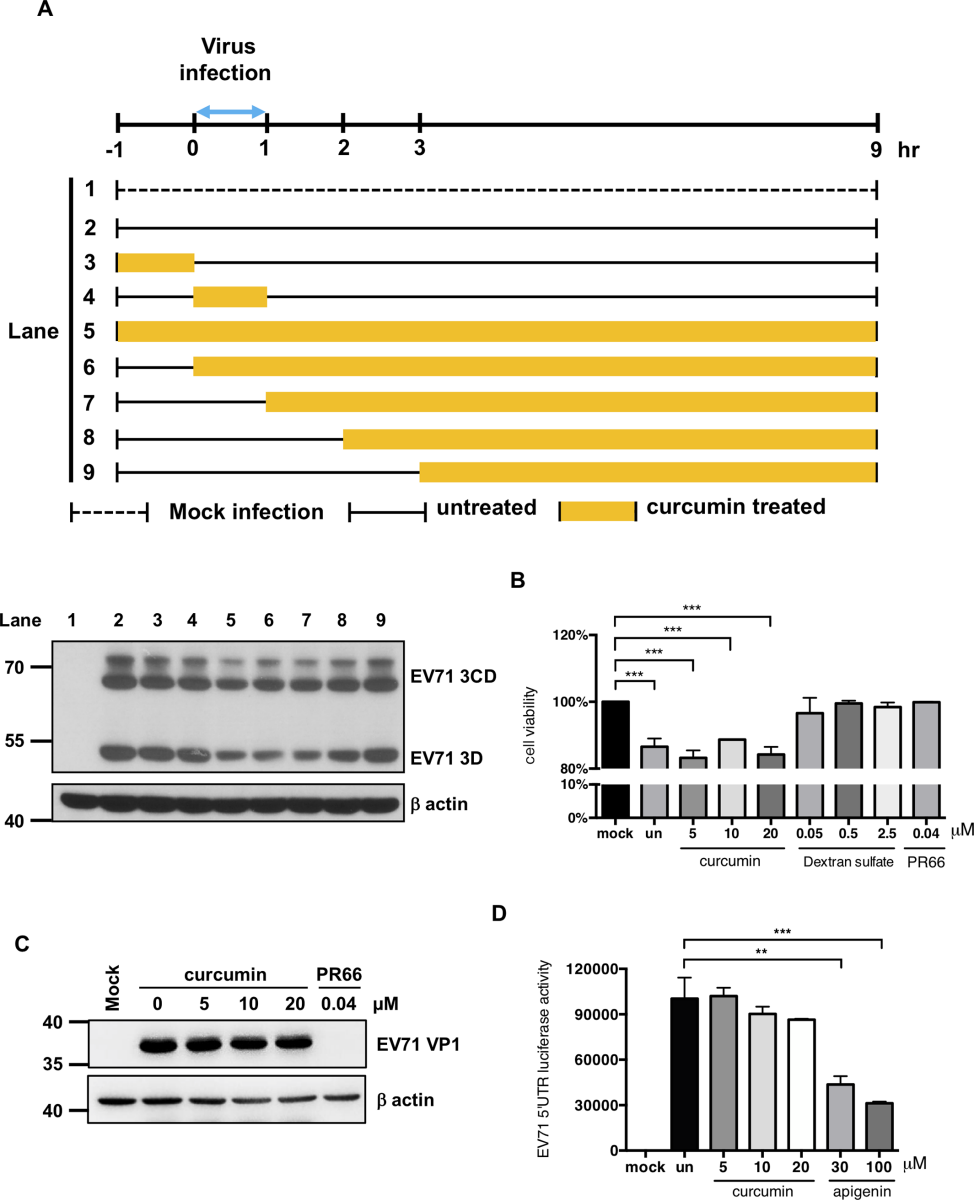
Curcumin inhibits EV71 protein expression when added at the early stages of infection. (A) A time-of-addition assay was performed to assess the anti-EV71 mechanism of curcumin. HT29 cells were seeded on culture plates and infected with EV71 at the MOI of 1. Viral adsorption was allowed to occur from 0 to 1 hour p.i. Curcumin (10 μM) was added to the cells at the indicated times. After treatment, the cells were washed three times, and the medium was replaced. After 9 hours of infection, the cells were harvested, and total protein was isolated for Western blot analysis. An anti-EV71 3D antibody was used to detect the viral protein expression level. Expression of β-actin was used as an internal control. (B) To test whether curcumin can modulate the virus adsorption to the cells, EV71 virus infected cells in the absence or presence of curcumin, dextran sulfate or PR66 for one hour on ice. And cell survival rates were determined at 48 hours p.i. by MTT assay. (C) To test whether curcumin can modulate the EV71 viral particles binding to cells, EV71 were incubated with different concentrations of curcumin, or PR66 at 0.04 μM for 1 hour on ice and then used to infect the HT29 cells. Total protein was extracted at 12 hours p.i. and subsequently subjected for Western blot to examine the expression levels of viral protein VP1 and host β-actin. (D) To test whether curcumin could modulate the activity of EV71 5’UTR IRES, in vitro translation assay was used to detect the EV71 5’UTR IRES luciferase activity in the absence or presence of curcumin or apigenin. Un: untreated.

To test whether curcumin is toxic to virus, EV71 was mixed with 5, 10 and 20 μM curcumin for 1 hour in room temperature and then infected cells. Cells infected with curcumin-treated virus particles expressed similar amounts of viral proteins as cells infected with untreated virus (S4A Fig). These data indicated that curcumin was not viricidal to EV71. A time-of-addition assay was performed and revealed that pretreatment with curcumin had no effect on viral protein expression. We found that incubating the cells with curcumin during the adsorption period was essential to observing the antiviral effects of curcumin (Fig 4A). These data indicated that curcumin functions during the early steps of the virus cycle. To further examine whether curcumin affects the adsorption process, curcumin was added to cells at the same time as virus and then removed after 1 hour of adsorption on ice. After incubation for 48 hours, the cellular viability was determined. No difference was observed between curcumin-treated cells and control cells. In contrast to curcumin, dextran sulfate and PR66 significantly interfered with virus adsorption (Fig 4B). Thus, the anti-EV71 activity of curcumin is not mediated by an effect on virus adsorption.

To further examine whether curcumin can affect the binding of EV71 to intestinal epithelial cells, EV71 was treated with curcumin at 4°C for one hour and then used to infect cells at the MOI of 1 at 37°C for an additional hour. Total protein samples were harvested at 12 hours p.i. and used for Western blotting to analyze viral protein expression. As shown in Fig 4C, there was no significant difference between cells treated with curcumin and those treated with medium alone. In contrast to curcumin, PR66 significantly interfered with binding of EV71 to cells. Thus, curcumin had no influence on the attachment of EV71 viral particles to cells. Previous studies revealed that the anti-EV71 activities of phytochemicals such as kaempferol and sorafenib are related to their ability to down-regulate IRES activity [16, 17]. To test whether curcumin can affect IRES activity, an *in vitro* IRES activity assay was performed. EV71 5′UTR-firefly luciferase RNA was incubated with lysates collected from intestinal epithelial cells treated with or without curcumin, and the IRES activity was determined by measuring the firefly luciferase activities. EV71 IRES activity was not affected by curcumin treatment, in contrast to the effect observed with apigenin (Fig 4D).

### Curcumin up-regulates Erk phosphorylation in intestinal epithelial cells

**Fig 5:**
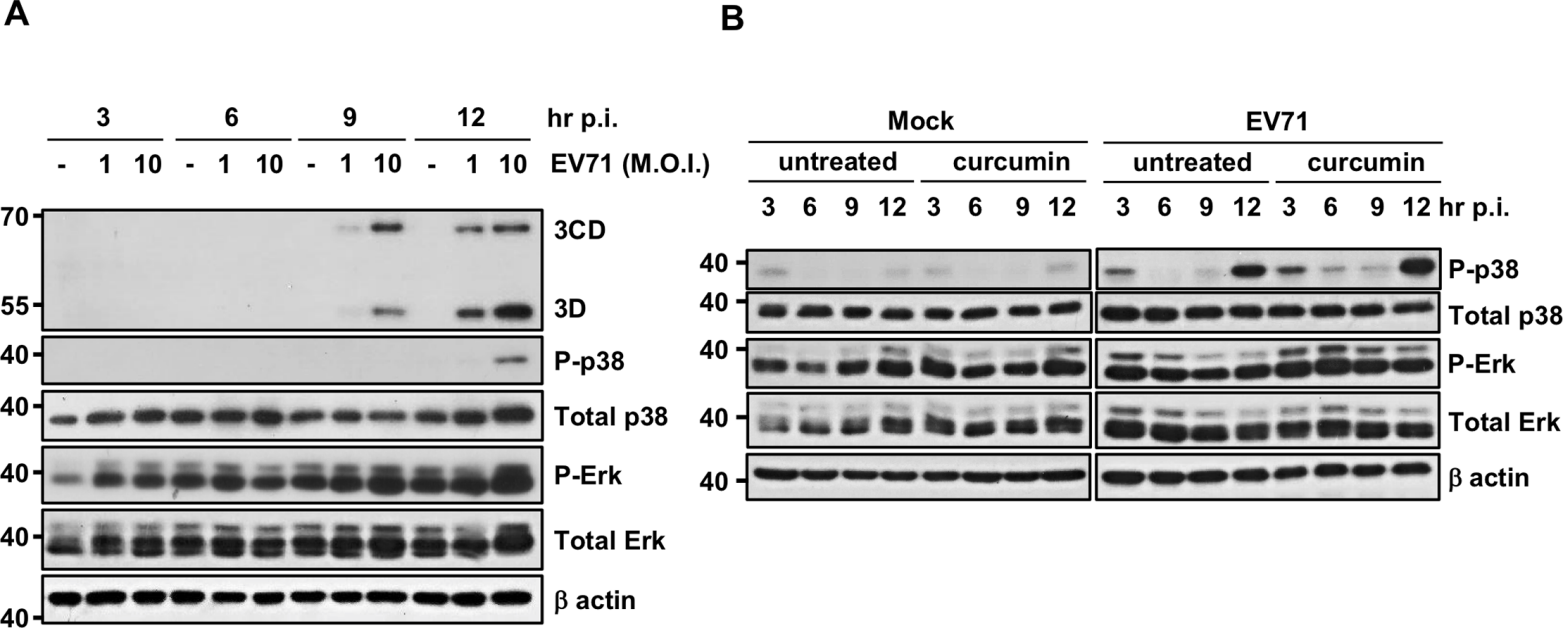
Curcumin does not suppress EV71 by modifying MAPK pathways. (A) Cells were infected with EV71 at different MOIs. Total cell lysates were harvested at different time points, and Erk and p38 phosphorylation was detected by immunoblot analysis. (B) To detect the effect of curcumin on MAPK pathways, HT29 cells were seeded in plates and infected with EV71 at the MOI of 1 with or without 10 μM curcumin in the medium. The cells were harvested at different time points, and total protein was isolated for Western blot analysis. Anti-P-Erk, anti-P-p38, anti-Total Erk, anti-Total p38, and anti-EV71 3D antibodies were applied to detect the phosphorylation of kinases.

Signaling transduction pathways could be affected by virus infection. As shown in Fig 5A, Erk activation was detected at 3 hours p.i with EV71, while p38 phosphorylation occurred at 12 hours p.i. in intestinal epithelial cells. However, our experimental results revealed that curcumin treatment further increased Erk and p38 phosphorylation in EV71-infected cells (Fig 5B). A previous study showed that activation of the mitogen-activated protein kinases (MAPKs) Erk and p38 facilitated EV71 replication [18]. Therefore, increased phosphorylation of these two kinases may up-regulate viral protein translation. Thus, the influence of curcumin on the Erk and p38 signaling pathways is not involved in its anti-EV71 effects. In addition, curcumin treatment has no influence on JNK and c-Jun phosphorylation in these cells (S5 Fig).

### Curcumin-induced alteration of PKCδ phosphorylation in intestinal epithelial cells

**Fig 6:**
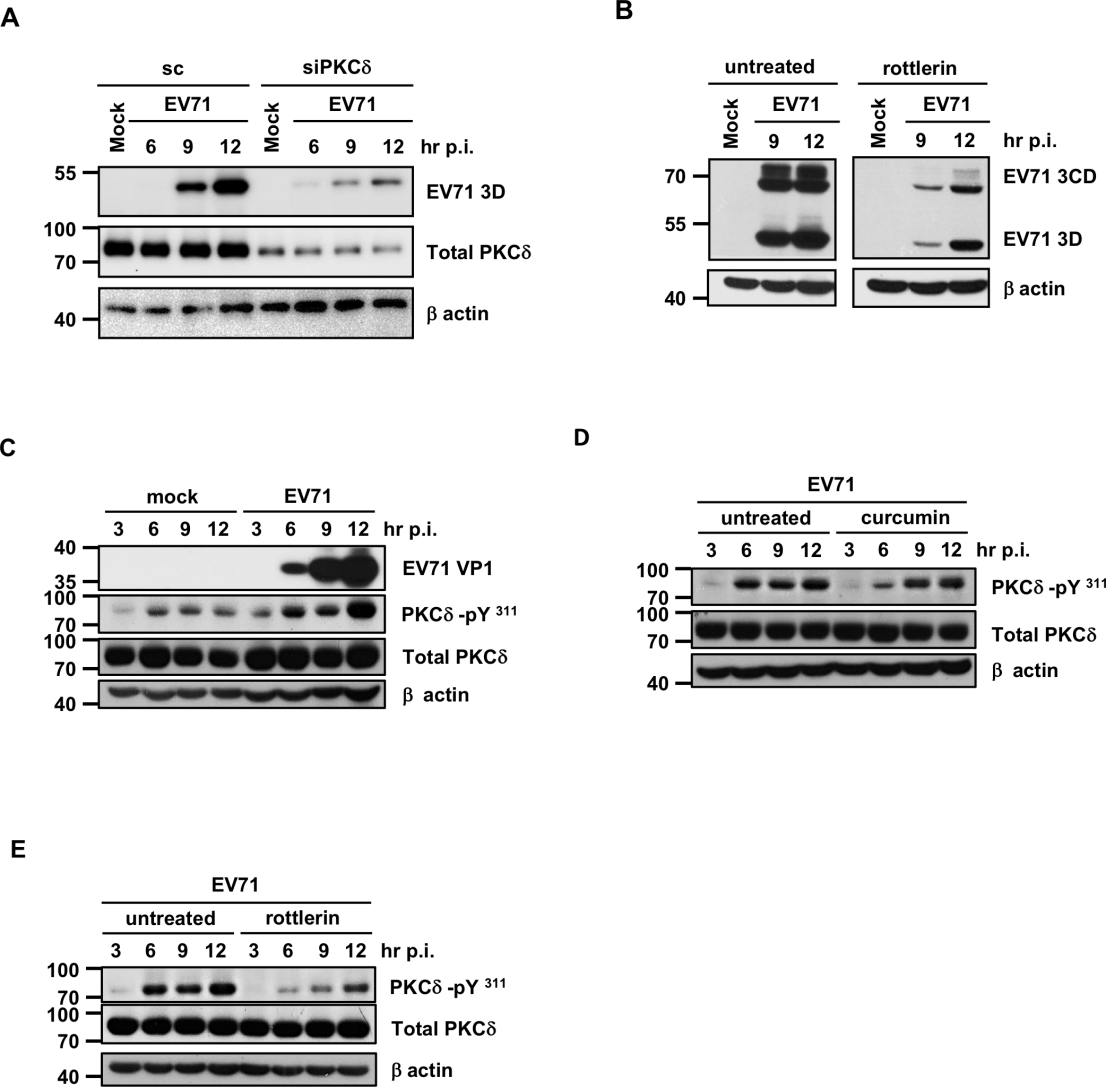
Curcumin inhibits EV71 infection-induced PKCδ phosphorylation. (A) PKCδ siRNA and scramble siRNA were transfected into HT29 cells for 24 hours, and the cells were then infected with EV71 at the MOI of 1. Total protein samples were isolated at different time points and subjected to Western blot analysis to examine the expression levels of PKCδ and EV71 viral protein 3D. (B) HT29 cells were seeded and infected with EV71 in the presence of rottlerin (5 μM), a known PKCδ inhibitor. The expression levels of viral protein 3D and β-actin were then analyzed by Western blot. (C) Protein samples were harvested from mock-infected and EV71-infected HT29 cells at various time points. Immunoblotting was performed to examine the expression levels of viral protein VP1, total PKCδ, P-PKCδ (Tyr311) and β-actin. (D) Total protein was extracted from EV71-infected HT29 cells in the presence or absence of 10 μM curcumin. The expression of PKCδ, P-PKCδ (Tyr311), EV71 3D, and β-actin was analyzed by Western blot. (E) Cells were treated with rottlerin and infected with EV71. Western blot analysis was performed to detect the expression levels of P-PKCδ (Tyr311), Total PKCδ and β-actin.

Curcumin is a pleiotropic molecule that affects numerous targets. Curcumin suppresses the activation of transcription factors such as NF-κB and AP-1 and inhibits the activities of several enzymes, including cytochrome P450s [19]. PKCδ activation has been shown to play important roles in the replication of several viruses [20]. Furthermore, curcumin has been shown to affect PKCδ phosphorylation, thus impairing the activation of this enzyme [21]. To understand whether PKCδ is involved in EV71 replication, PKCδ siRNA was transfected into cells, and viral protein expression levels were examined. The efficiency of gene knockdown was confirmed. Our results showed a drastic decrease in viral protein expression in PKCδ-knockdown cells (Fig 6A). Thus, PKCδ plays an essential role in EV71 infection. Furthermore, rottlerin, a PKCδ inhibitor, effectively reduced viral protein translation in HT29 cells (Fig 6B) [22]. Thus, we hypothesized that PKCδ phosphorylation may be affected by EV71 and contribute to viral growth. The expression levels of phosphorylated PKCδ were examined in EV71-infected HT29 cells, and our results showed that P-PKCδ (Tyr311) was up-regulated (Fig 6C). Interestingly, curcumin treatment affected the phosphorylation of PKCδ at Tyr311 at 6 hours p.i. (Fig 6D). The rottlerin-mediated decrease in Tyr311 phosphorylation was also observed in EV71-infected HT29 cells (Fig 6E).

### Curcumin treatment inhibits EV71 viral protein expression in differentiated intestinal epithelial cells

**Fig 7:**
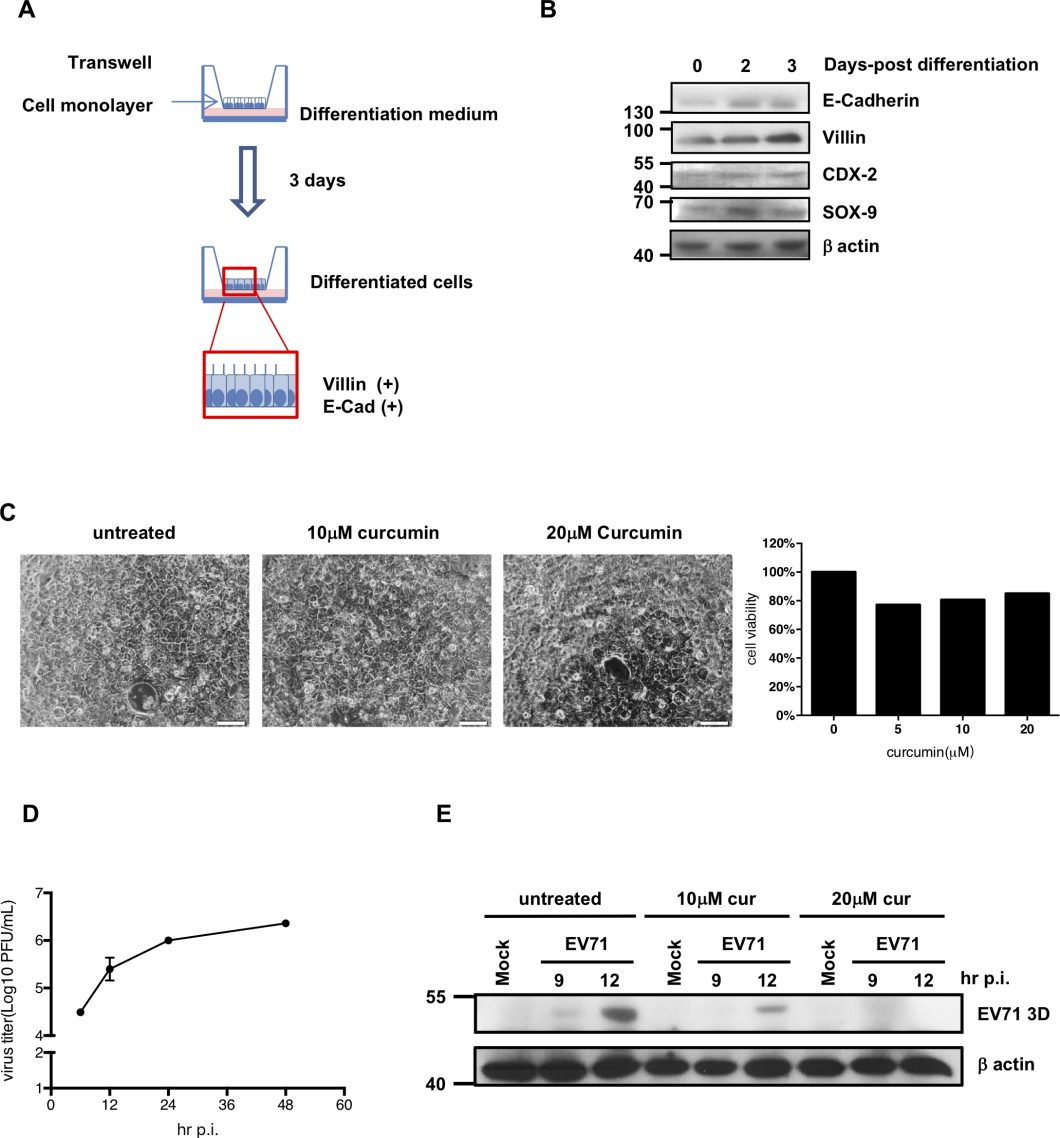
Curcumin treatment suppresses EV71 translation in differentiated C2BBe1 cells. (A) C2BBel cells were seeded on transwell plates and cultivated in enterocyte differentiation medium for 3 days. Total protein was isolated from cells at different differentiation time points. (B) Western blot analysis was performed to measure the expression levels of E-cadherin, Villin, CDX-2, and SOX-9. The expression of β-actin was used as an internal control. (C) Differentiated C2BBe1 cells were treated with 10 and 20 μM curcumin for 48 hours and the morphologies were observed using inverted microscope (Magnification = 200x). Trypan blue was added and the cell viabilities were counted. (D) Differentiated C2BBe1 cells were seeded and infected with EV71 at the MOI of 2. Total cell lysates were collected at different time points and subjected to plaque assays to determine the viral titers. (E) Differentiated C2BBe1 cells were infected with EV71 at the MOI of 10 in the absence or presence of curcumin (10 and 20 μM). The cells were harvested at different time points for protein isolation. The expression levels of viral protein 3D were measured by Western blot analysis.

To further prove that curcumin functions as an anti-EV71 agent in intestinal epithelial cells, we tested the antiviral activity of curcumin using differentiated C2BBe1 cells. C2BBe1 cells are derived from Caco-2 human colon epithelial carcinoma cells. Interestingly, these cells can be induced to differentiate into mature intestinal epithelial cells and have been used in studies of their physiological properties [23]. We first incubated C2BBe1 cells in differentiation medium for 3 days (Fig 7A). Differentiation was then confirmed by Western blot analysis. The expression levels of E-cadherin and villin increased, while the expression of SOX-9 increased at first and then declined (Fig 7B). The cytotoxicity of curcumin has been measured and the results revealed that no significant cell death was observed (Fig 7C). These differentiated cells were susceptible to EV71 infection and supported active replication of the virus (Fig 7D). To test whether curcumin exerts anti-EV71 effects, differentiated cells were treated with curcumin and infected with EV71, and protein samples were then extracted to measure viral protein expression levels (Fig 7E). Viral protein expression was decreased in differentiated cells treated with curcumin.

## Discussion

EV71 is one of the major viral pathogens spread via the fecal-oral route. The virus enters the human body through contact with contaminated feces or hands, and the gastrointestinal tract has been identified as the primary infection site. Previous studies have shown that drugs and probiotics can be used to treat intestinal epithelial cells infected with gut pathogens. For example, pretreatment of intestinal epithelial cell lines with probiotics protected against the deleterious effects of enteroinvasive *Escherichia coli* [24]. Furthermore, the ingestion of green tea polyphenols has been shown to inhibit the growth of *Helicobacter pylori* and reduce the associated inflammation [25]. Phytochemicals are considered safe treatment options because no serious adverse effects have been reported following the administration of these plant-derived compounds, and animal studies have demonstrated that many of these substances are well tolerated. Therefore, phytochemicals may be used to treat various pathological conditions. Thus, the identification of herbs or drugs that work directly on intestinal epithelial cells may be useful in treating EV71 infection.

Several phytochemicals, such as oblongifolin M, kaempferol, baicalin, and apigenin, have been demonstrated to act as antiviral agents against EV71 infection [16, 26, 27, 28]. Furthermore, naturally occurring compounds such as *Kalanchoe gracilis* leaf extract and chebulagic acid isolated from *Terminalia chebula* have been reported to exhibit anti-EV71 activity following intraperitoneal injection [29, 5]. However, no phytochemical has been examined for its ability to inhibit EV71 replication in intestinal epithelial cells. Curcumin, a phytochemical isolated from *Curcuma longa* Linn, is a flavonoid with anti-inflammatory, antioxidant, and cancer preventative properties. It has long been used as a spice in Southern Asia and the Middle East. Recent studies have shown that curcumin is effective in treating inflammatory diseases such as rheumatoid arthritis and inflammatory bowel disease [30, 31]. Curcumin treatment also protects intestinal epithelial cells from bacterial invasion [32]. Previous studies demonstrated that curcumin suppresses the replication of multiple viruses, including CVB3, hepatitis C, and herpes simplex [33]. Our findings demonstrate that curcumin is able to inhibit EV71 replication in intestinal epithelial cells. In addition, curcumin also inhibits the replication of CVB3 and EVD68. Thus, curcumin could be used to treat various enteroviruses.

The results of our time-of-addition assay suggest that curcumin affects the early steps of EV71 viral replication. Although curcumin impairs the binding of HCV to primary human hepatocytes, our results showed that curcumin pretreatment does not affect the binding of EV71 to intestinal epithelial cells [34]. Several flavonoids are known to exert their antiviral effects by regulating IRES activity [16, 17]. Therefore, we examined whether curcumin affected viral protein translation by modulating IRES activity. However, our reporter assays showed that curcumin does not affect the IRES activity of EV71.

Curcumin interferes with the binding and fusion of hepatitis C virus in primary human hepatocytes, while other studies have shown that the induction of heme oxygenase-1 and suppression of AKT are also involved [34, 35]. As curcumin has begun to be studied in more detail, additional antiviral effects of this compound have been identified. For example, the abilities of curcumin to down-regulate VP16-mediated recruitment of RNA polymerase II and to dysregulate the ubiquitin proteasome system are responsible for suppressing replication of HSV and CVB3, respectively [14,33]. These observations indicate that several mechanisms are involved in the antiviral activity of curcumin.

Several signaling pathways are activated during EV71 infection. Previous studies showed that EV71 infection activates the Erk and p38 signaling pathways, which are essential for EV71 replication [36,37]. Inhibitors of these signaling proteins have been shown to efficiently block EV71 infection [18, 38]. Our data confirmed that these two pathways were also up-regulated in EV71-infected intestinal epithelial cells. However, curcumin treatment also activates the Erk and p38 pathways in these cells. Thus, the ability of curcumin to modulate these signaling pathways cannot explain its anti-EV71 activity.

In addition to MAPKs, curcumin has been demonstrated to modulate the activity of various protein kinases [39, 40]. In addition to maintaining physiological homeostasis, PKC has been implicated in the replication of dengue virus [41, 42]. Furthermore, PKCδ affects HIV replication via modulation of the post-entry step [43]. However, whether PKCδ is related to picornavirus growth is unclear. Our data revealed that knockdown of PKCδ expression suppresses the translation of EV71, and thus PKCδ may also play a role in EV71 replication. However, we did not observe changes in PKCδ expression in EV71-infected intestinal epithelial cells. Surprisingly, our results showed that EV71 infection induces phosphorylation of PKCδ Y311 and that this phosphorylation is reduced in curcumin-treated cells. The biological functions of PKC are regulated by phosphorylation at specific serine/threonine sites, which affects the catalytic activity and spatial distribution of the protein [44]. Previous studies revealed that Y311 phosphorylation results in conformational changes and thus regulates PKCδ actions [45]. To our knowledge, this is the first report demonstrating that virus infection induces the phosphorylation of PKCδ. In addition to curcumin, rottlerin, a known PKCδ inhibitor, also down-regulates EV71-induced phosphorylation of PKCδ Y311 during the early stage of infection. Addition of rottlerin also results in decreased viral protein expression. Therefore, EV71 induces PKCδ phosphorylation in intestinal epithelial cells, and down-regulation of the expression levels of total PKCδ or PKCδ-pY311 suppresses viral protein expression. According to Ueda’s paper, PKCδ can activate the ERK1 and MEK1 in fibroblast-like cells [46]. However, in addition to PKCδ, other stimulations are involved with the activation of the Erk and MAPK pathways. For example, the activation of Ras may result in the Erk phosphorylation [47]. Furthermore, a previous paper demonstrated that curcumin is able to induce the activation of Ras/Erk signaling pathway in gastric carcinoma cells [48]. Thus, curcumin may activate the Erk pathway in a PKCδ-independent way.

Compared with undifferentiated carcinoma cell lines, differentiated cells express proteins indicative of mature enterocytes and possess characteristic physiological intestinal epithelial properties, including nutrient and drug adsorption and barrier function [49, 50]. To further confirm that curcumin is effective in suppressing EV71 in normal human intestinal epithelial cells, differentiated C2BBe1 cells were used. These cells have been used to study host cell-microbe interactions owing to their ability to form a polarized monolayer [51]. Our results proved that curcumin also efficiently inhibited viral translation in these differentiated cells.

In summary, this study demonstrated that curcumin exhibits anti-EV71 activity in intestinal epithelial cells. The antiviral activity of this compound is not related to viral binding, adsorption, or alteration of viral IRES activity. Our results show that curcumin may function as a PKCδ inhibitor by reducing the phosphorylation of a specific tyrosine residue in intestinal epithelial cells. Furthermore, we demonstrate that the dysregulation of PKCδ inhibits EV71 replication. Thus, the ability of curcumin to suppress PKCδ activation may contribute to its anti-EV71 activity.

## Supporting information

S1 Fig EV71 infection induces the activation of caspase-3. HT-29 cells were infected with EV71 at the MOI of 1 and 10 for 24 hours. The cells were then fixed and immunostained with anti-EV71 3D and anti-active caspase 3 mAbs. Dylight 594 and 488 conjugated secondary antibodies were then applied to interact with the bound antibodies. DAPI was used for counterstain the cell nuclei (Magnification = 200x).
(TIFF)

S2 Fig PR66 treatment suppresses EV71 replication. PR66 has been reported that can inhibit EV71 replication, so it was served as positive control in this paper. (A) The treatment condition of PR66 in HT-29 was the same with curcumin. To determine the cytotoxicity of PR66, HT-29 cells were seeded and treated with various concentration of PR66 for 48 hours and MTT assay was used to determine the cellular viability. (B) HT-29 cells were seeded and infected with EV71 at the MOI of 1 in the absence or presence of PR66 (0.04μM). Total cell lysates were collected at 9 hours p.i. and subjected for plaque assay to determine the viral titers. (C) Cells were harvested at 9 hours p.i. and total protein was extracted to determine the expression of EV71 VP1 protein by Western blot. The expression of β-actin was used as internal control. (D) RT-qPCR analysis was performed to detect the amounts of viral RNA.
(TIFF)

S3 Fig Curcumin suppresses the replication of CVB3 and EVD68 in intestinal epithelial cells. HT29 cells were treated with 10 μM curcumin and then infected with CVB3 and EVD68 at the MOI of 1. The cell lysates were harvested at 12 and 24 hours p.i. and the virus titers were determined using plaque assay.
(TIFF)

S4 Fig Curcumin lacks of the virucidal activity. To test whether curcumin can destroy the EV71 viral particles, EV71 viral particles were incubated with various concentration of curcumin in room temperature for 1hour and then used to infected HT-29 cells. Total protein was extracted at 12 hours p.i. and determined the expression of EV71 VP1 protein by Western blot. The expression of β-actin was used as internal control.
(TIFF)

S5 Fig Curcumin does not affect the phosphorylation of JNK and c-Jun. To detect the effect of curcumin in phosphorylation of JNK and c-Jun, HT-29 cells were seeded in plates and infected by EV71 at the MOI of 1 in absence or presence of curcumin. Cells were harvested at different time point and total protein was collected for western blot analysis. Anti-p-JNK, anti-p-c-Jun and anti-EV71 3D antibodies were applied to detect the phosphorylation status of JNK and c-Jun. The expression of β-actin was used as internal control.
(TIFF)

S1 File Minimal manuscript dataset. (ZIP)
