# Supplementary figures and images for: Inhibition of EV71 by curcumin in intestinal epithelial cells
Source: PLoS One. 2018 Jan 25;13(1):e0191617. doi: 10.1371/journal.pone.0191617 (PMC5784943; doi:10.1371/journal.pone.0191617)

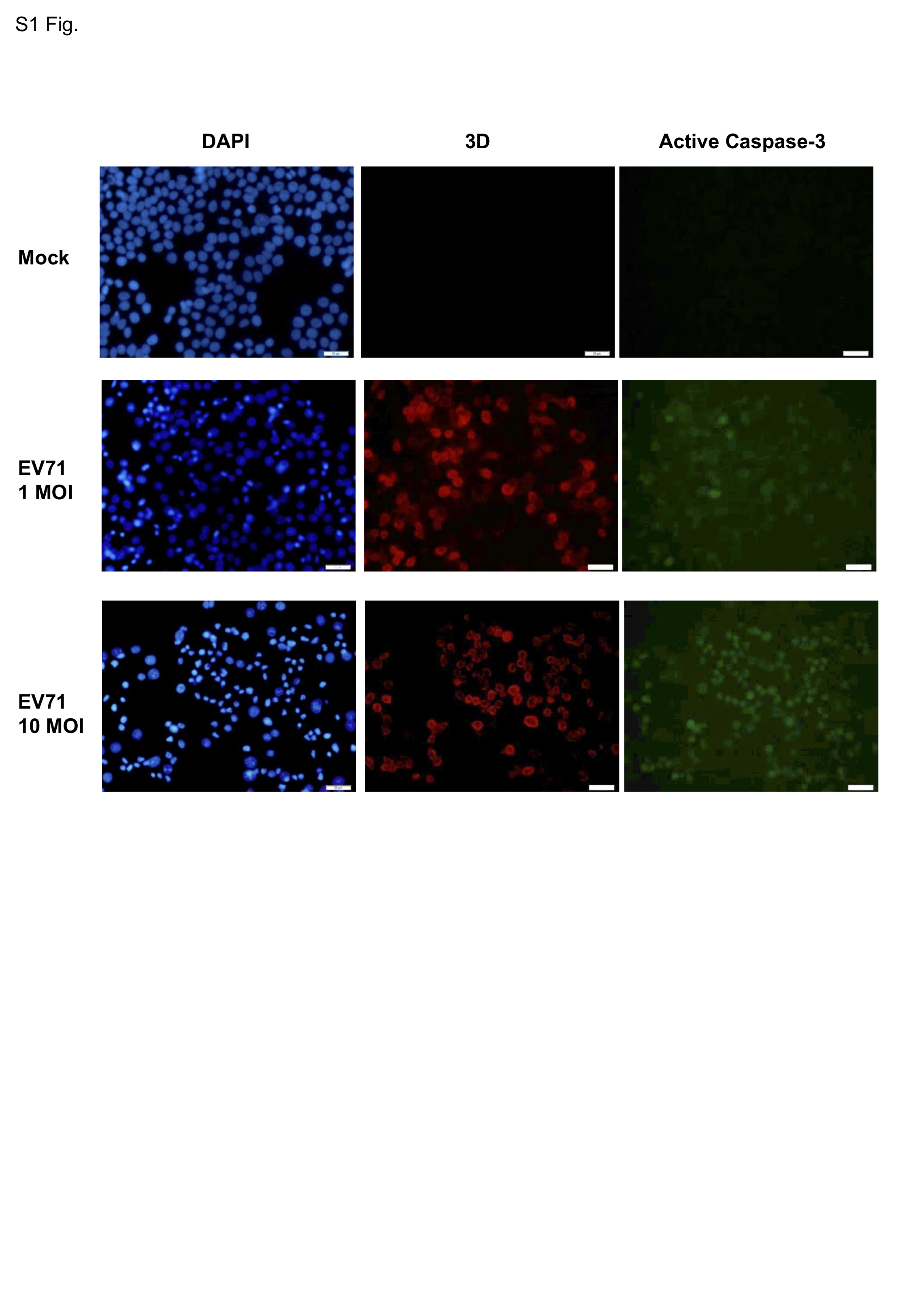

Supplement: S1 Fig — HT-29 cells were infected with EV71 at the MOI of 1 and 10 for 24 hours. The cells were then fixed and immunostained with anti-EV71 3D and anti-active caspase 3 mAbs. Dylight 594 and 488 conjugated secondary antibodies were then applied to interact with the bound antibodies. DAPI was used for counterstain the cell nuclei (Magnification = 200x). (TIFF) [file pone.0191617.s001.tiff]

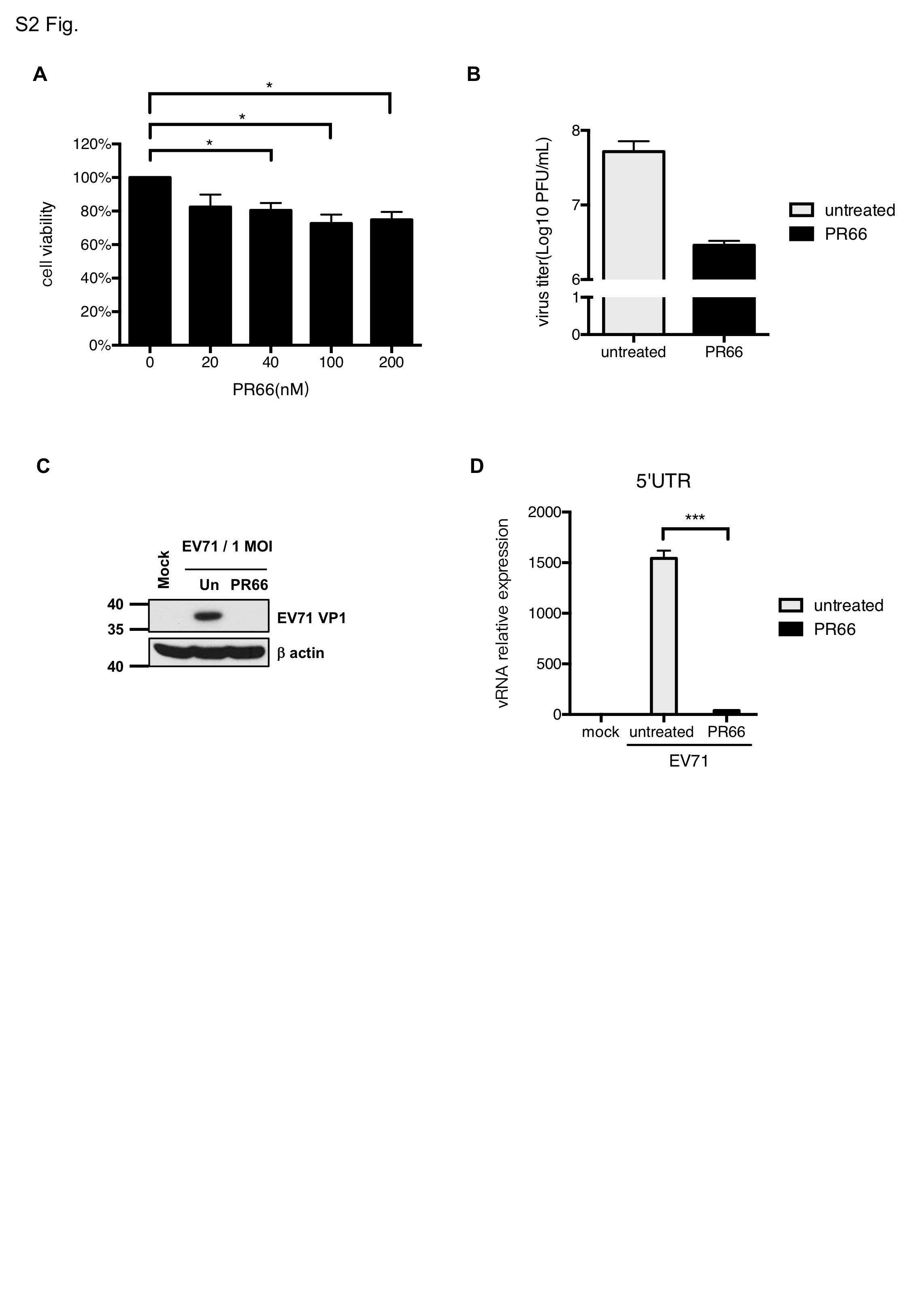

Supplement: S2 Fig — PR66 has been reported that can inhibit EV71 replication, so it was served as positive control in this paper. (A) The treatment condition of PR66 in HT-29 was the same with curcumin. To determine the cytotoxicity of PR66, HT-29 cells were seeded and treated with various concentration of PR66 for 48 hours and MTT assay was used to determine the cellular viability. (B) HT-29 cells were seeded and infected with EV71 at the MOI of 1 in the absence or presence of PR66 (0.04μM). Total cell lysates were collected at 9 hours p.i. and subjected for plaque assay to determine the viral titers. (C) Cells were harvested at 9 hours p.i. and total protein was extracted to determine the expression of EV71 VP1 protein by Western blot. The expression of β-actin was used as internal control. (D) RT-qPCR analysis was performed to detect the amounts of viral RNA. (TIFF) [file pone.0191617.s002.tiff]

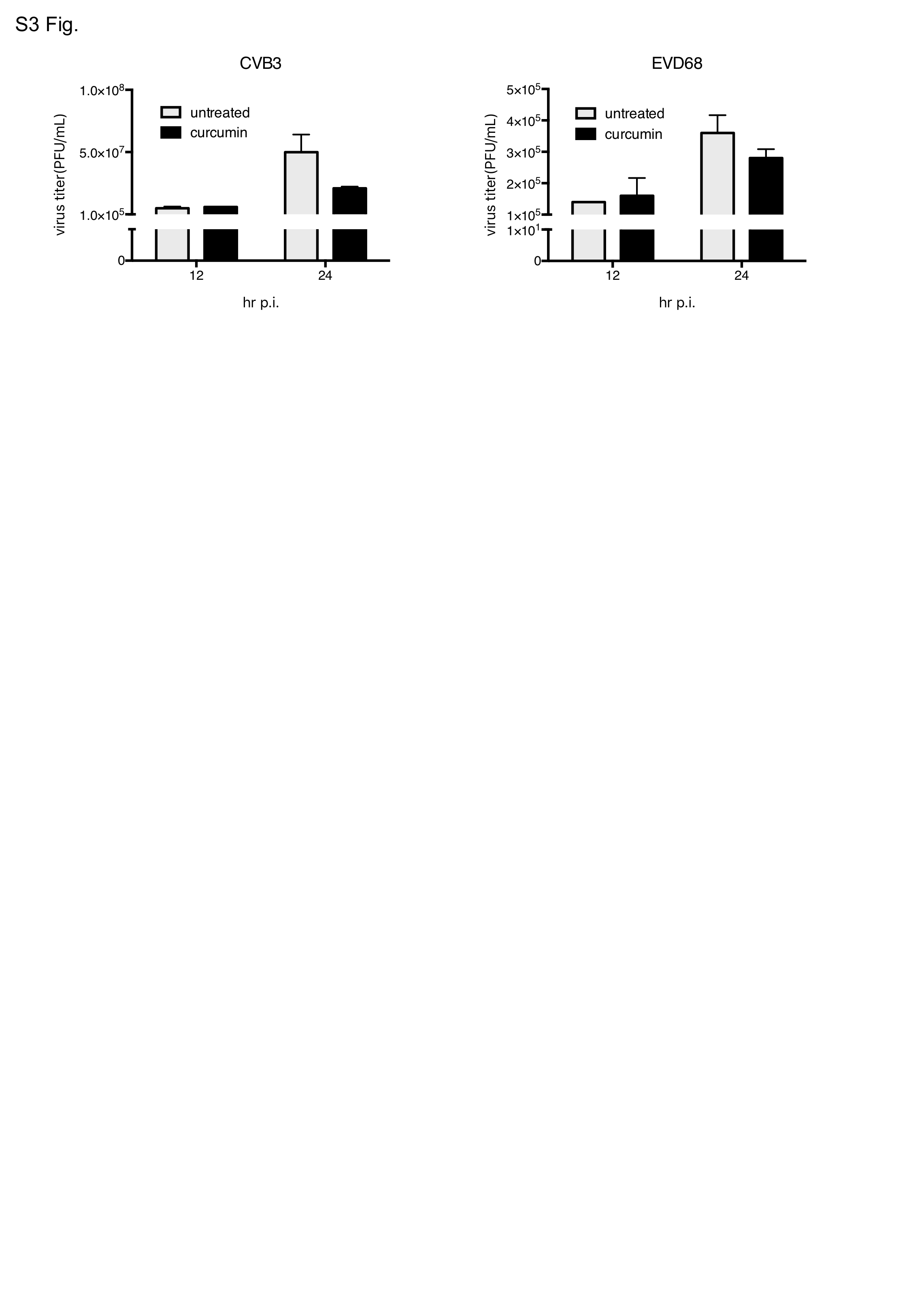

Supplement: S3 Fig — HT29 cells were treated with 10 μM curcumin and then infected with CVB3 and EVD68 at the MOI of 1. The cell lysates were harvested at 12 and 24 hours p.i. and the virus titers were determined using plaque assay. (TIFF) [file pone.0191617.s003.tiff]

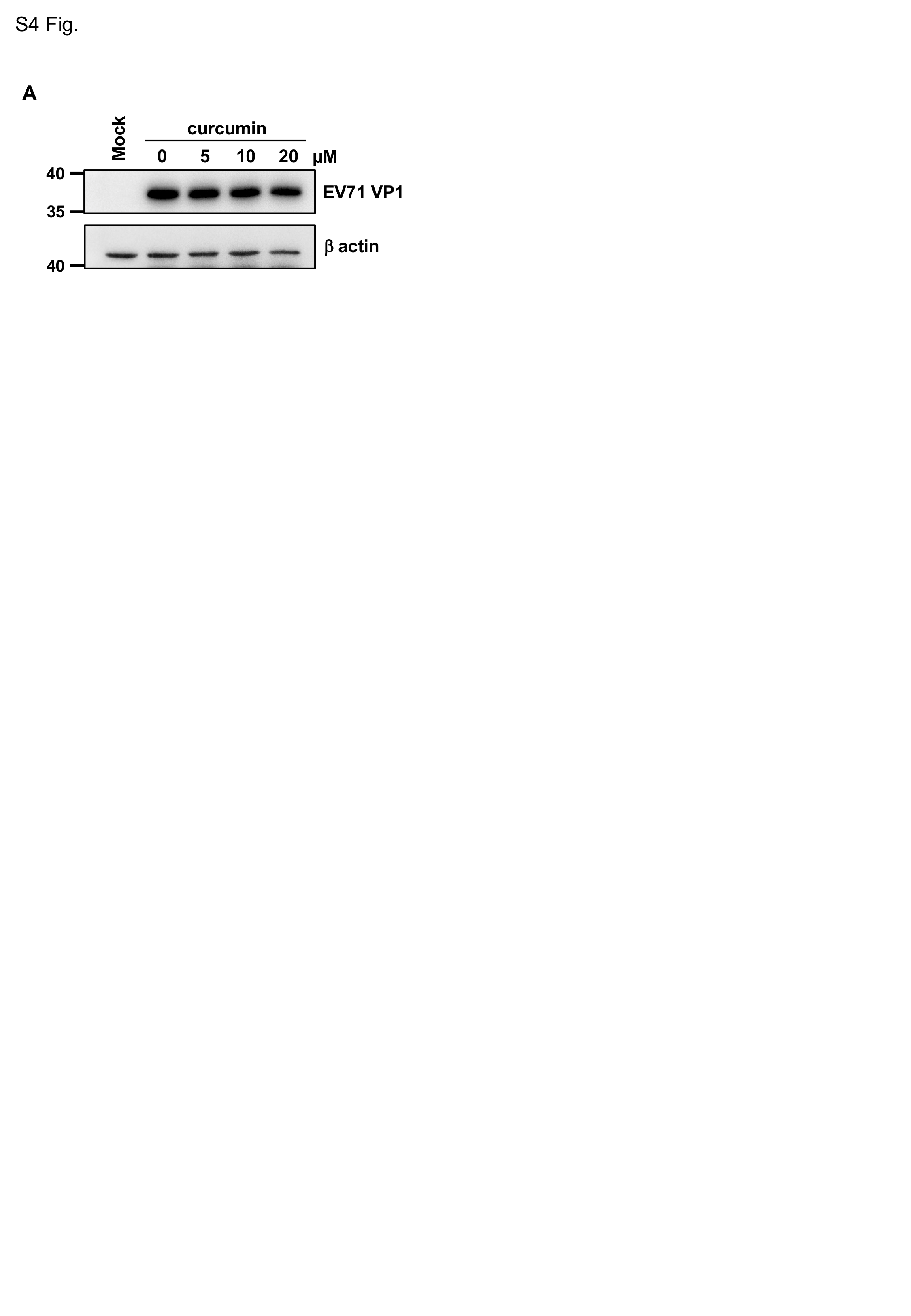

Supplement: S4 Fig — To test whether curcumin can destroy the EV71 viral particles, EV71 viral particles were incubated with various concentration of curcumin in room temperature for 1hour and then used to infected HT-29 cells. Total protein was extracted at 12 hours p.i. and determined the expression of EV71 VP1 protein by Western blot. The expression of β-actin was used as internal control. (TIFF) [file pone.0191617.s004.tiff]

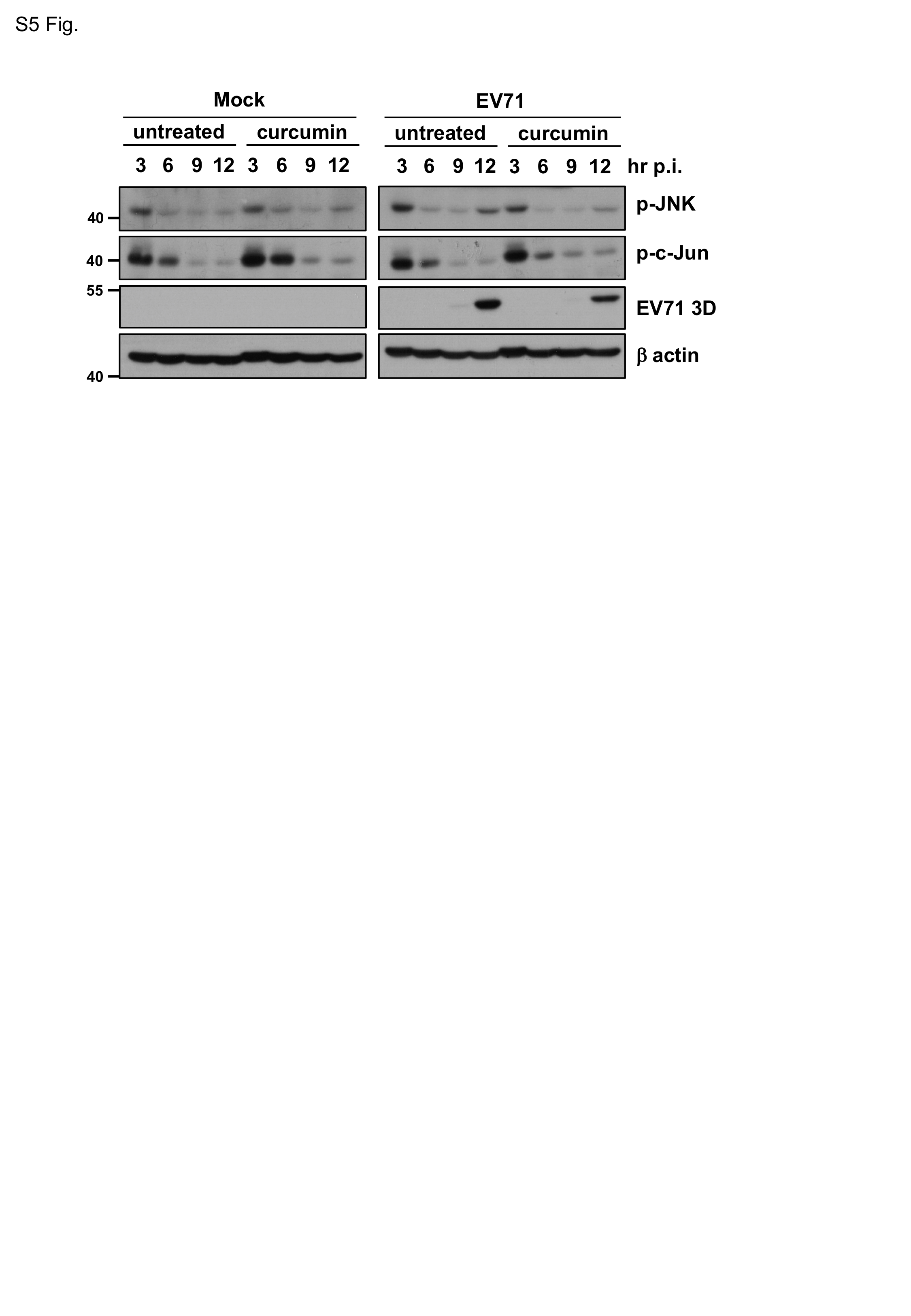

Supplement: S5 Fig — To detect the effect of curcumin in phosphorylation of JNK and c-Jun, HT-29 cells were seeded in plates and infected by EV71 at the MOI of 1 in absence or presence of curcumin. Cells were harvested at different time point and total protein was collected for western blot analysis. Anti-p-JNK, anti-p-c-Jun and anti-EV71 3D antibodies were applied to detect the phosphorylation status of JNK and c-Jun. The expression of β-actin was used as internal control. (TIFF) [file pone.0191617.s005.tiff]

**Fig 1. HT-29 cells are permissive to EV71 infection.**

(A)

mock


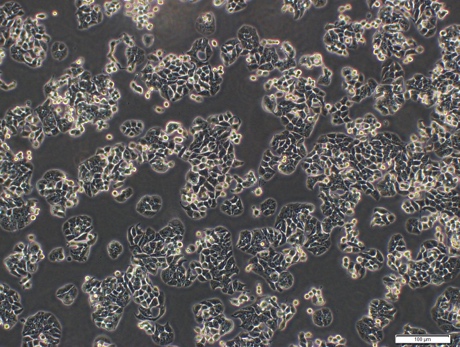

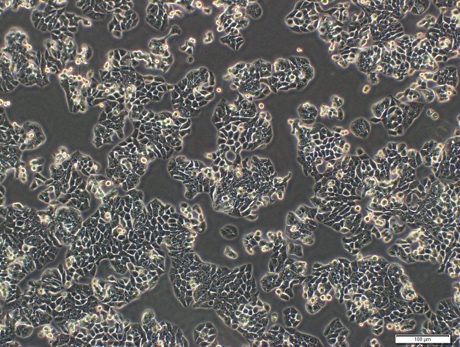


12hr 1 MOI EV71


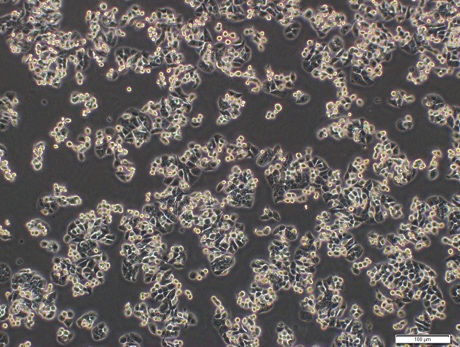

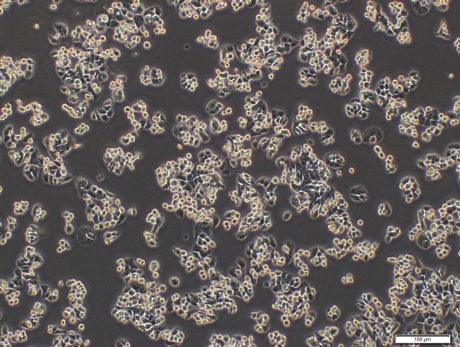


24hr 1MOI EV71


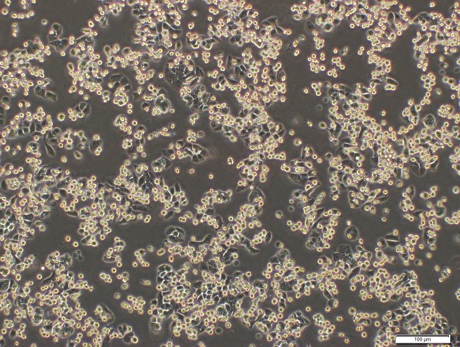

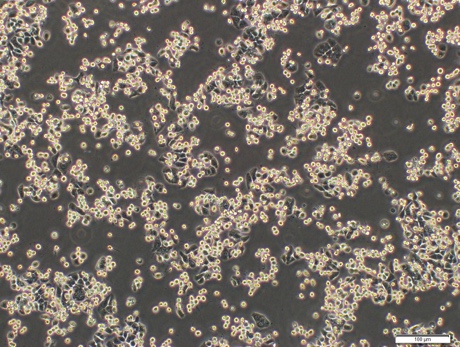


(B)

mock


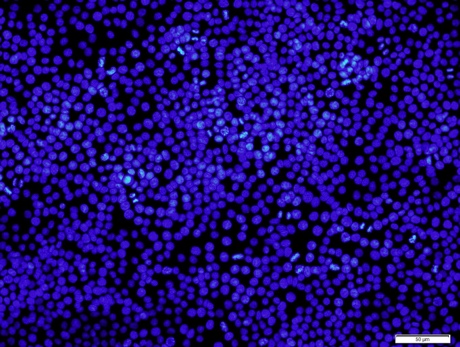


1MOI EV71 12hr


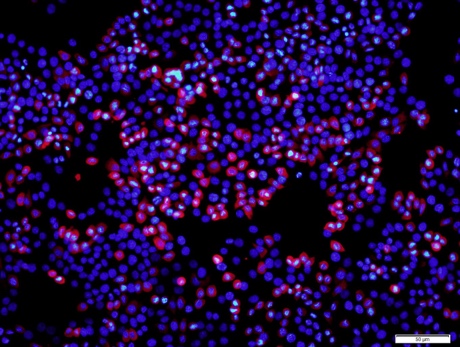

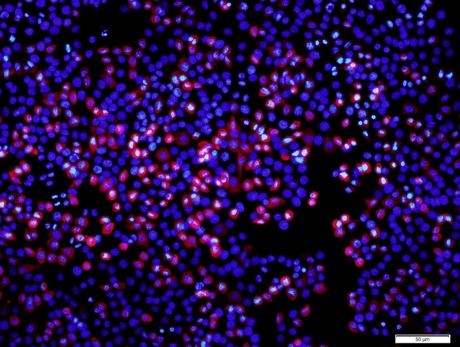


1MOI EV71 24hr


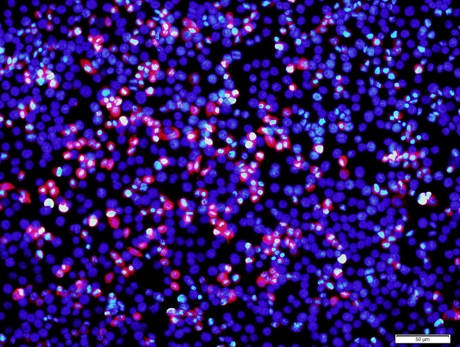

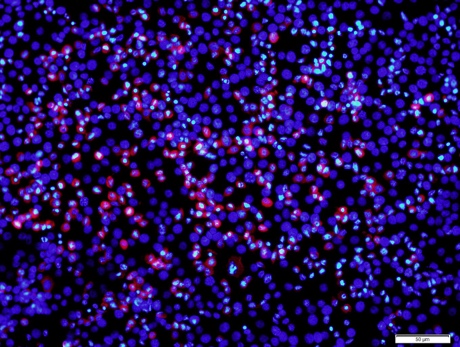


(C)

(D)

Supplement: S1 File — (ZIP) [file pone.0191617.s006.zip › Minimal manuscript dataset/Fig 1.docx]

**Fig 2. Curcumin cytotoxicity assay.**

(A)

0μM


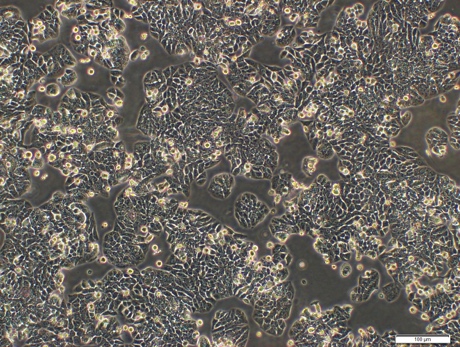

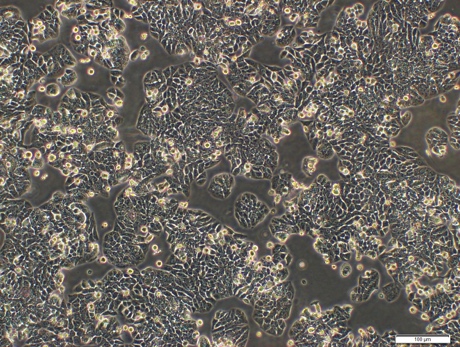


5μM


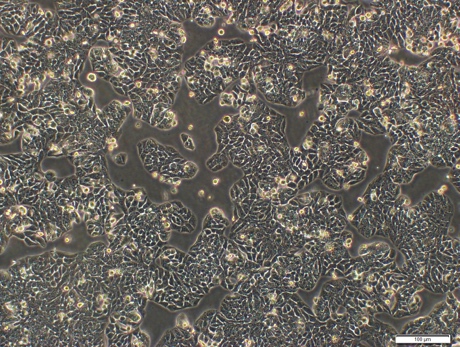

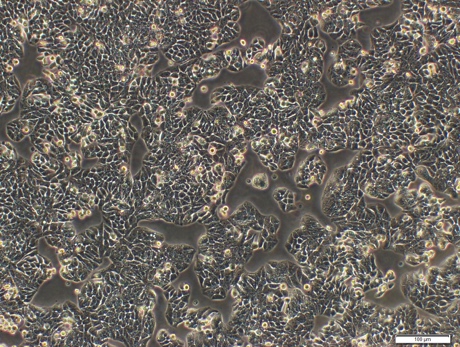


10μM


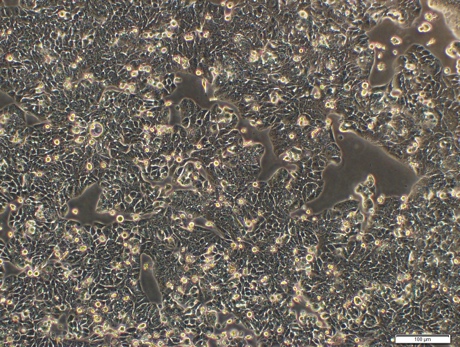

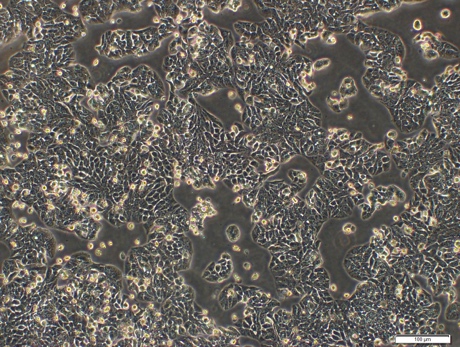


20μM


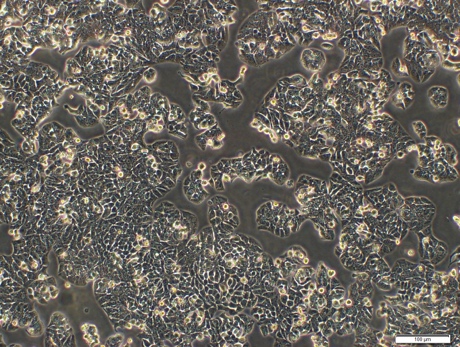

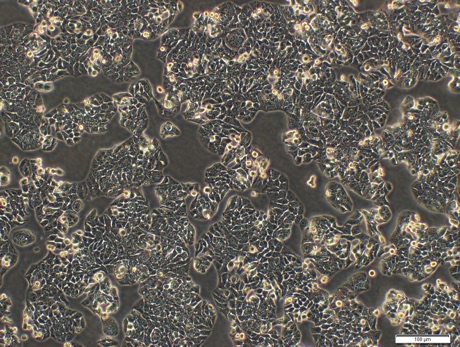


40μM


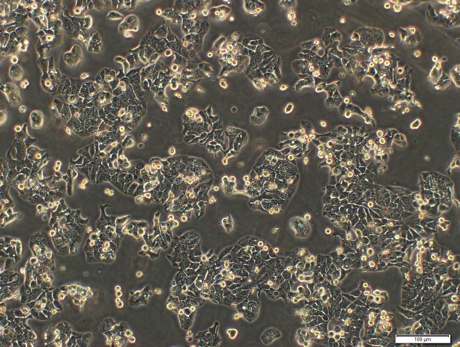

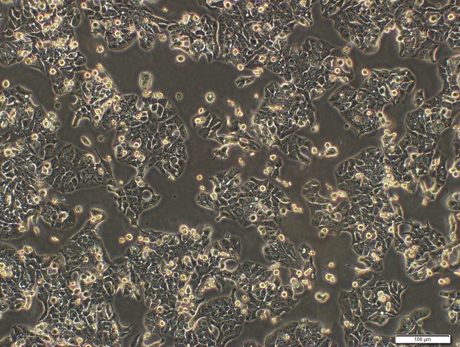


50μM


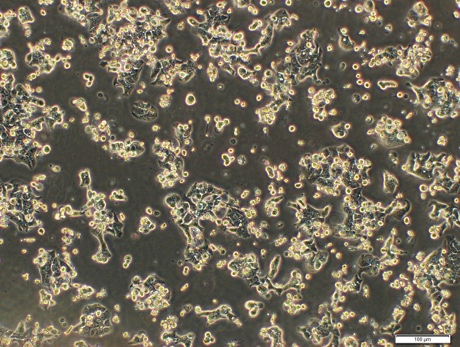

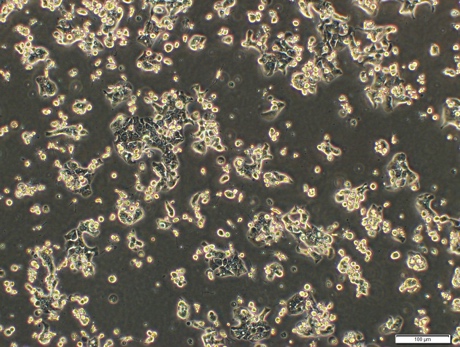


(B)

(C)

Supplement: S1 File — (ZIP) [file pone.0191617.s006.zip › Minimal manuscript dataset/Fig 2.docx]
