# Supplementary material for: Inhibition of EV71 by curcumin in intestinal epithelial cells
Source: PLoS One. 2018 Jan 25;13(1):e0191617. doi: 10.1371/journal.pone.0191617 (PMC5784943; doi:10.1371/journal.pone.0191617)
Supplement: S1 File — (ZIP) [file pone.0191617.s006.zip › Minimal manuscript dataset/S1 Fig .docx]

**S1 Fig. EV71 infection induces the activation of caspase-3**.

Mock

DAPI EV71 3D


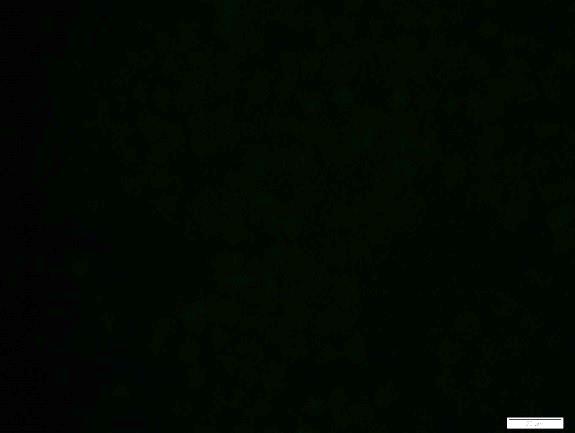

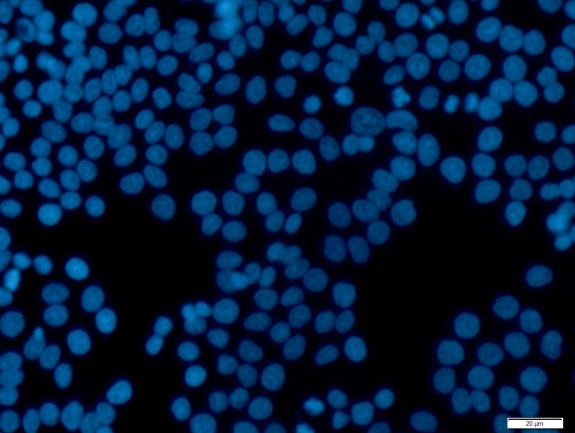


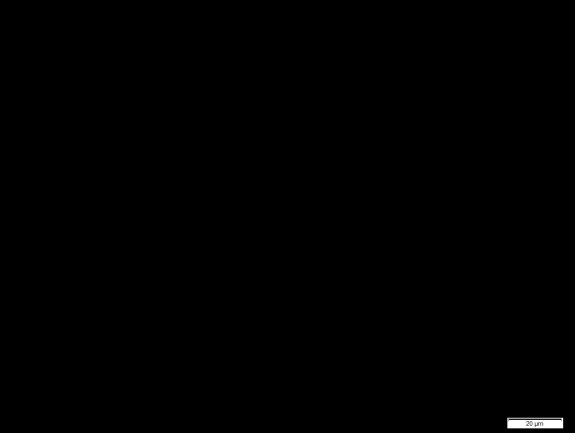
caspase 3

48hr 1MOI EV71

DAPI EV71 3D


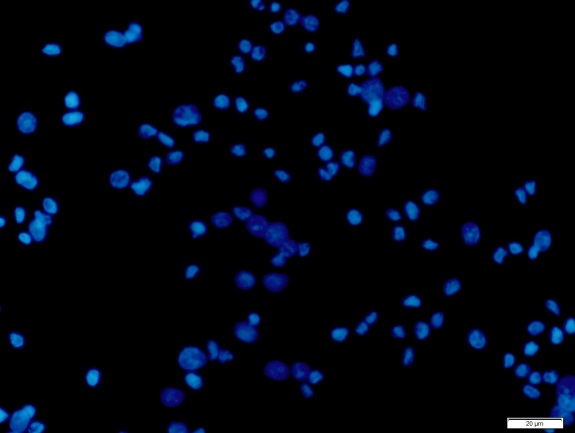

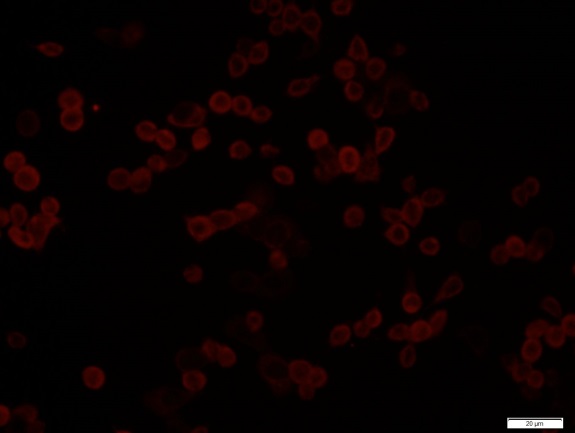


Caspase 3


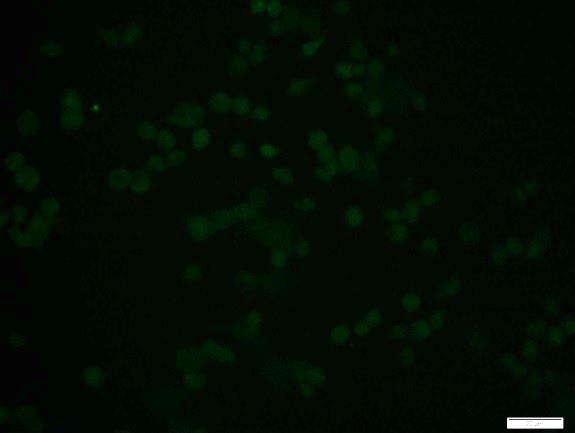


48hr 10MOI EV71

DAPI EV71 3D


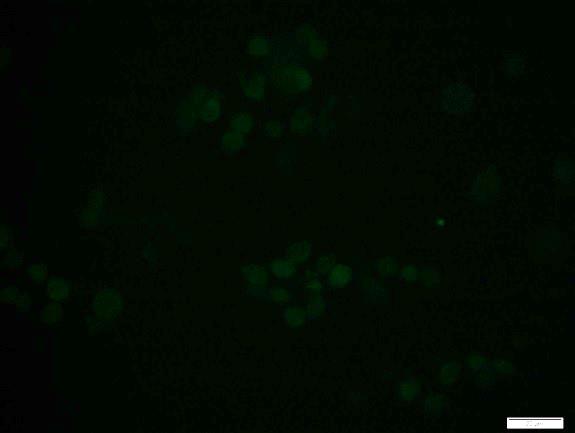

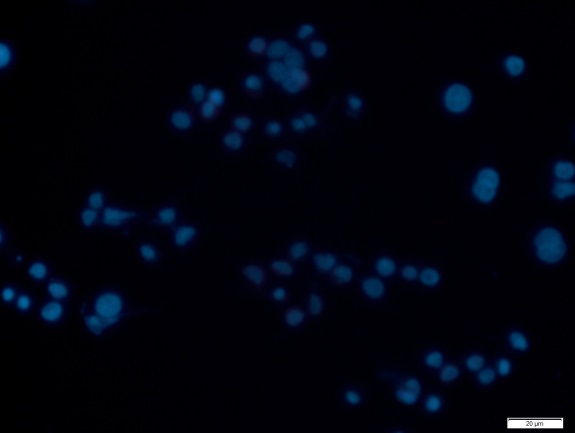

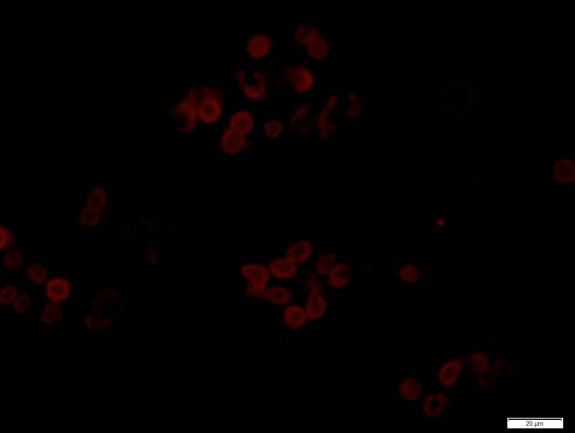


Caspase 3
