# Supplementary material for: Inhibition of EV71 by curcumin in intestinal epithelial cells
Source: PLoS One. 2018 Jan 25;13(1):e0191617. doi: 10.1371/journal.pone.0191617 (PMC5784943; doi:10.1371/journal.pone.0191617)
Supplement: S1 File — (ZIP) [file pone.0191617.s006.zip › Minimal manuscript dataset/S2 Fig.docx]

**S2 Fig.** **PR66 treatment suppresses EV71 replication.**

(A)

(B)

|  | 1M.O.I. 4643 infect PR66 treated HT29 - plaque assay | |
| --- | --- | --- |
|  | 1 | 2 |
| untreated | 66000000 | 38000000 |
| PR66 | 2600000 | 3180000 |

(C)


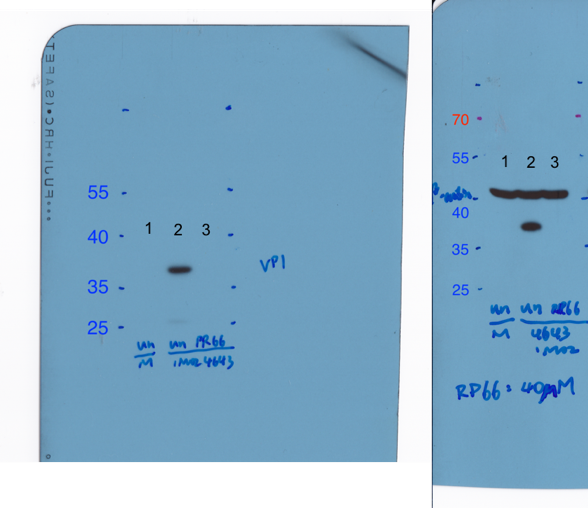


| Lane | sample |
| --- | --- |
| 1 | Un-treated, mock |
| 2 | Un-treated, EV71 |
| 3 | PR66, EV71 |

(D)
