# Supplementary material for: Inhibition of EV71 by curcumin in intestinal epithelial cells
Source: PLoS One. 2018 Jan 25;13(1):e0191617. doi: 10.1371/journal.pone.0191617 (PMC5784943; doi:10.1371/journal.pone.0191617)
Supplement: S1 File — (ZIP) [file pone.0191617.s006.zip › Minimal manuscript dataset/Fig 6.docx]

**Fig 6. Curcumin inhibits EV71 infection-induced PKCδ phosphorylation.**

(A)


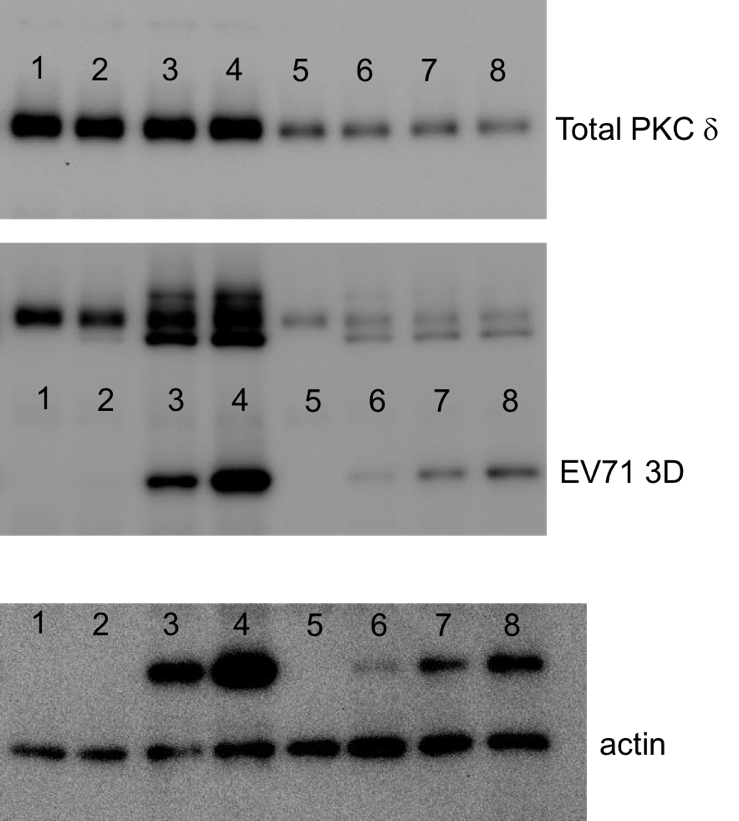


| Lane | sample |
| --- | --- |
| 1 | Sc transfectiom, mock |
| 2 | Sc transfection, EV71 ,6hr |
| 3 | Sc transfection, EV71 ,9hr |
| 4 | Sc transfection, EV71 ,12hr |
| 5 | siPKCδ transfection, mock |
| 6 | siPKCδ transfectio,EV71, 6hr |
| 7 | siPKCδ transfection, EV71, 9hr |
| 8 | siPKCδ transfection, EV71, 12hr |

(B)


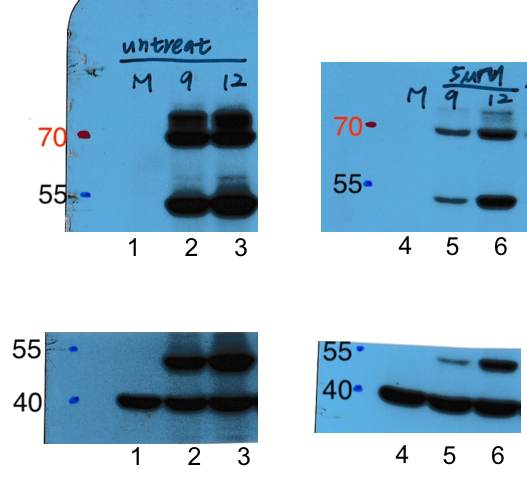


| Lane |  |
| --- | --- |
| 1 | Un-treated, mock |
| 2 | Un-treated, EV71, 9hr |
| 3 | Un-treated, EV71, 12hr |
| 4 | 5μM curcumin, mock |
| 5 | 5μM curcumin, EV71, 9hr |
| 6 | 5μM curcumin, EV71, 12hr |

(C)

| Lane | sample |
| --- | --- |
| 1 | Mock 3hr |
| 2 | Mock 6hr |
| 3 | Mock 9hr |
| 4 | Mock 12hr |
| 5 | 1MOI EV71 3hr |
| 6 | 1MOI EV71 6hr |
| 7 | 1MOI EV71 9hr |
| 8 | 1MOI EV71 12hr |


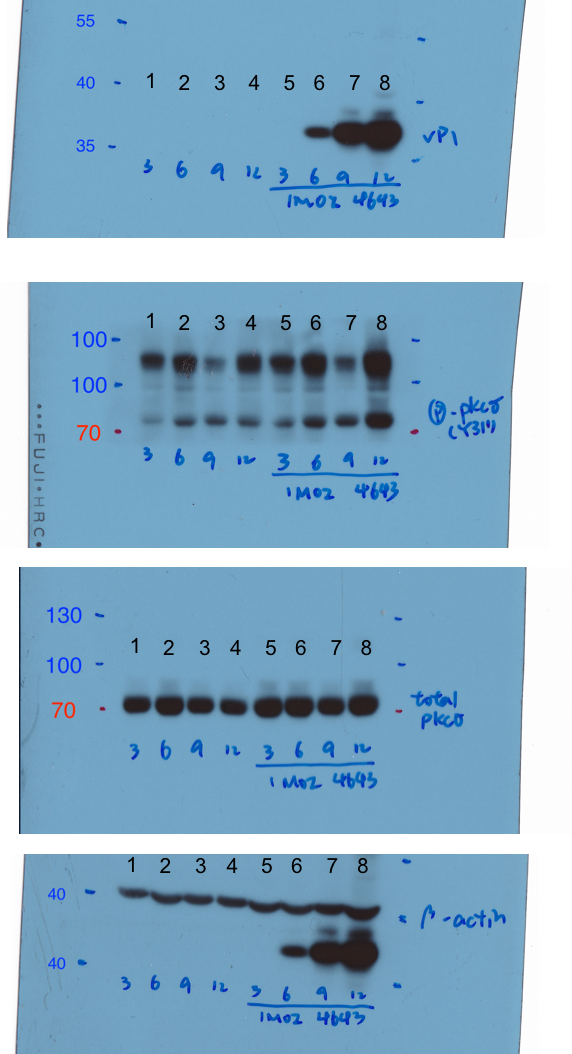


(D)


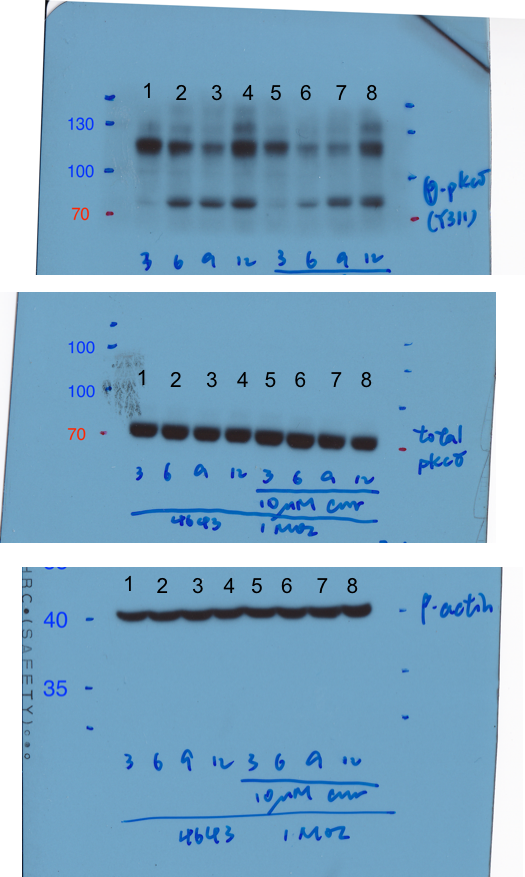


| Lane | sample |
| --- | --- |
| 1 | Un-treated ,1MOI EV71 3hr |
| 2 | Un-treated,1MOI EV71 6hr |
| 3 | Un-treated,1MOI EV71 9hr |
| 4 | Un-treated, 1MOI EV71 12hr |
| 5 | 10μM curcumin ,1MOI EV71 3hr |
| 6 | 10μM curcumin,1MOI EV71 6hr |
| 7 | 10μM curcumin,1MOI EV71 9hr |
| 8 | 10μM curcumin, 1MOI EV71 12hr |

(E)


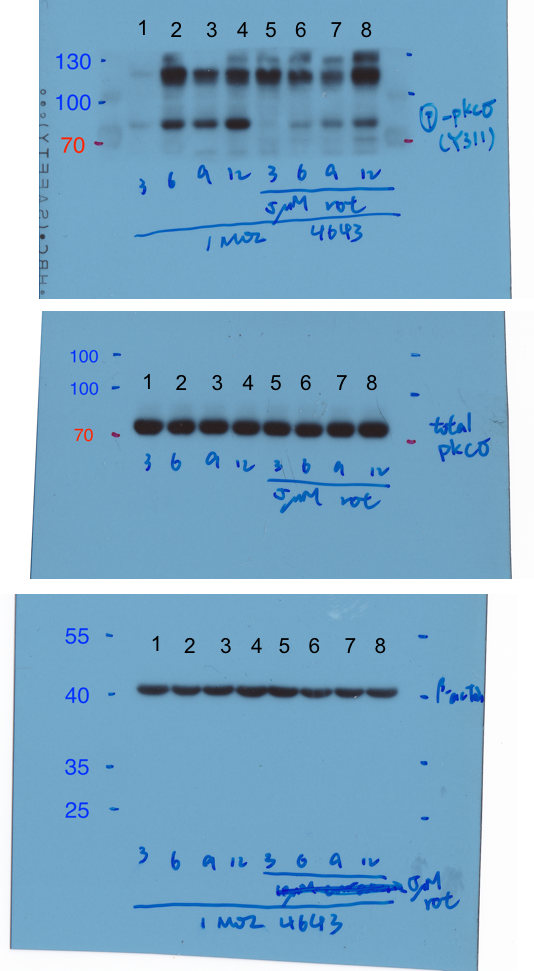


| Lane | sample |
| --- | --- |
| 1 | Un-treated ,1MOI EV71 3hr |
| 2 | Un-treated,1MOI EV71 6hr |
| 3 | Un-treated,1MOI EV71 9hr |
| 4 | Un-treated, 1MOI EV71 12hr |
| 5 | 5μM rottlerin ,1MOI EV71 3hr |
| 6 | 5μM rottlerin,1MOI EV71 6hr |
| 7 | 5μM rottlerin,1MOI EV71 9hr |
| 8 | 5μM rottlerin, 1MOI EV71 12hr |
