# Supplementary material for: Inhibition of EV71 by curcumin in intestinal epithelial cells
Source: PLoS One. 2018 Jan 25;13(1):e0191617. doi: 10.1371/journal.pone.0191617 (PMC5784943; doi:10.1371/journal.pone.0191617)
Supplement: S1 File — (ZIP) [file pone.0191617.s006.zip › Minimal manuscript dataset/Fig 7.docx]

**Fig 7**. **Curcumin treatment suppresses EV71 translation in differentiated C2BBe1 cells.**

(B)


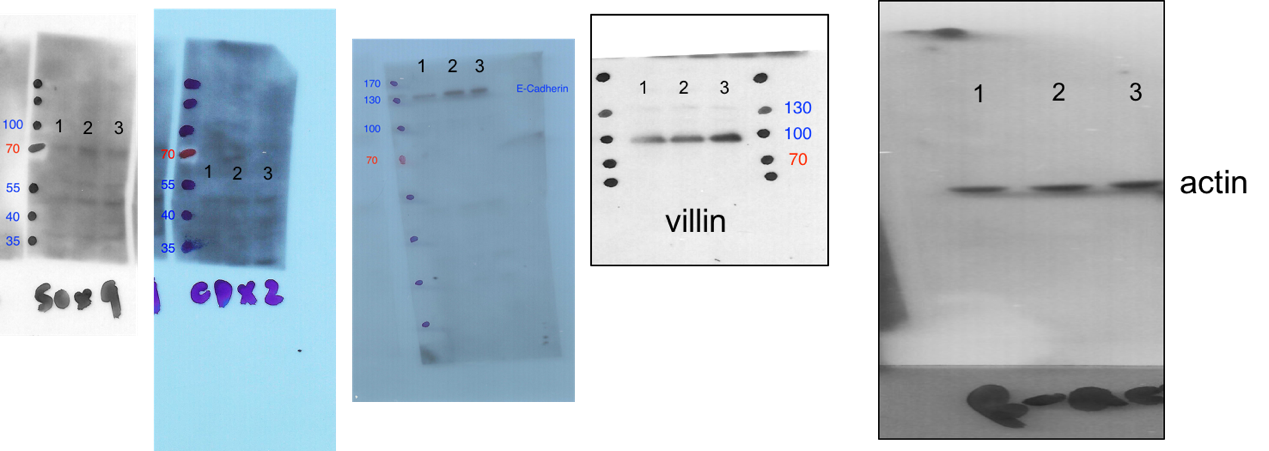


| Lane | sample |
| --- | --- |
| 1 | Differentiation 0 day |
| 2 | Differentiation 2 day |
| 3 | Differentiation 3 day |

(C)

un-treated


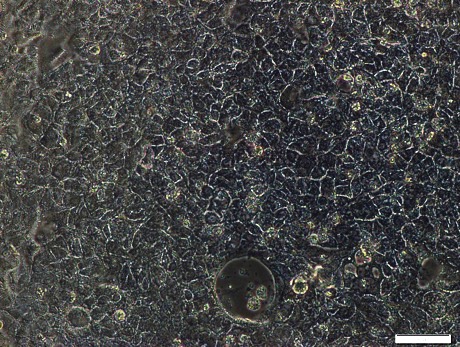

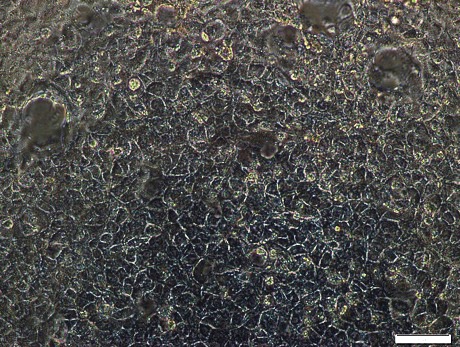


10μM curcumin


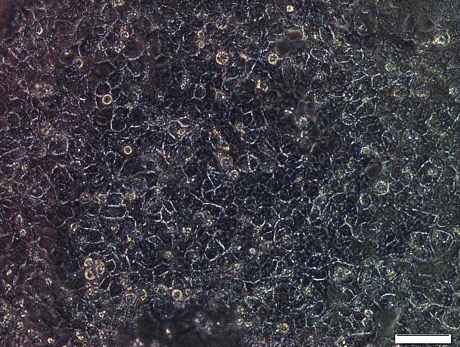

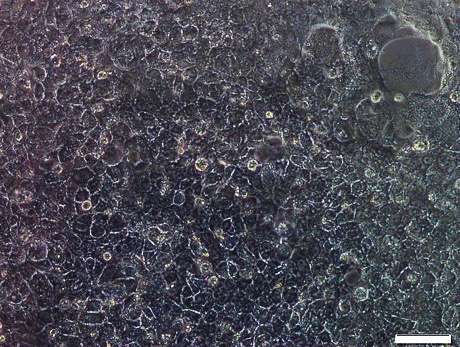


20μM curcumin


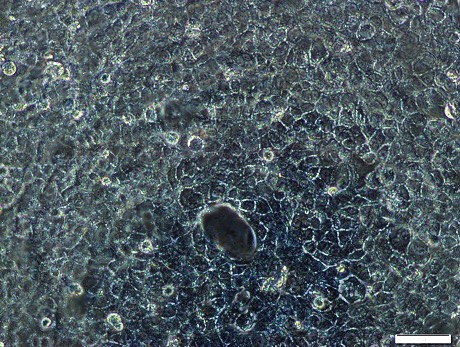

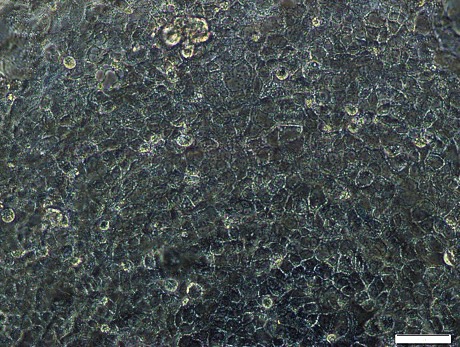


trypan blue

(D)

| Time | 1st | 2nd |
| --- | --- | --- |
| 6hr | 34000 | 28000 |
| 12hr | 120000 | 380000 |
| 24hr | 1000000 | 1000000 |
| 48hr | 2000000 | 2600000 |

(E)


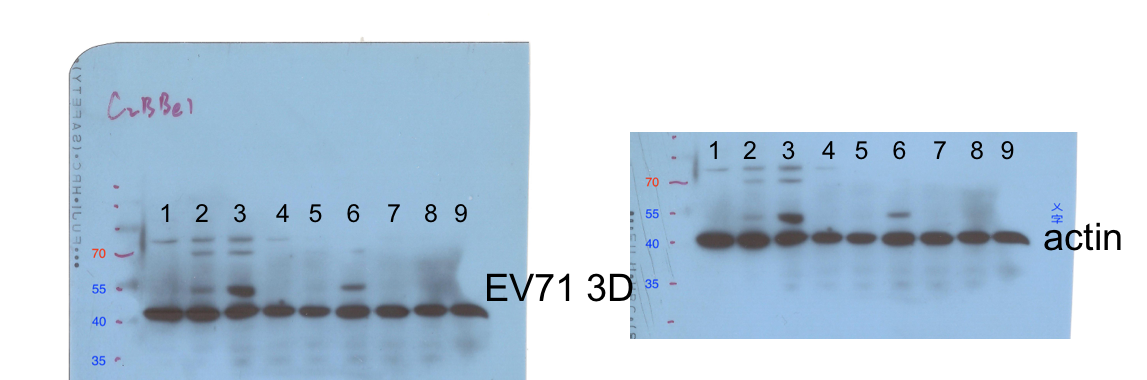


| Lane | sample |
| --- | --- |
| 1 | Un-treated, mock |
| 2 | Un-treated, EV71, 9hr |
| 3 | Un-treated, EV71, 12hr |
| 4 | 10μM curcumin, mock |
| 5 | 10μM curcumin, EV71, 9hr |
| 6 | 10μM curcumin, EV71, 12hr |
| 7 | 20μM curcumin, mock |
| 8 | 20μM curcumin, EV71, 9hr |
| 9 | 20μM curcumin, EV71, 12hr |
