# Supplementary material for: Inhibition of EV71 by curcumin in intestinal epithelial cells
Source: PLoS One. 2018 Jan 25;13(1):e0191617. doi: 10.1371/journal.pone.0191617 (PMC5784943; doi:10.1371/journal.pone.0191617)

**S5 Fig. Curcumin does not affect the phosphorylation of JNK and c-Jun.**
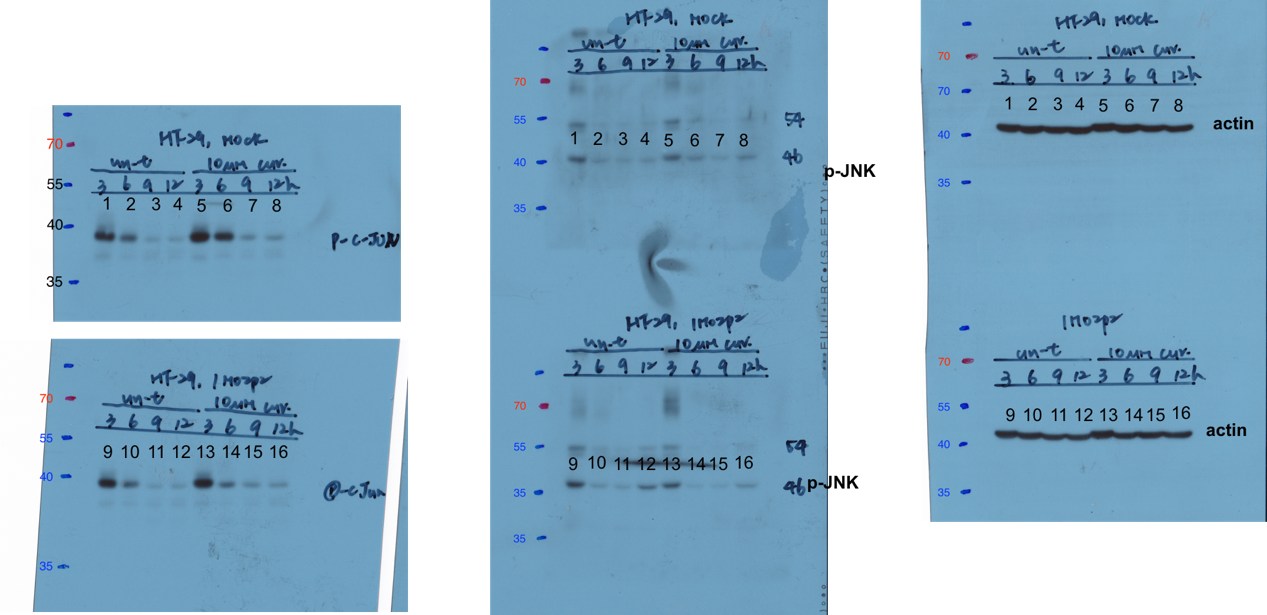


| Lane | sample |
| --- | --- |
| 1 | Un-treated, mock 3hr |
| 2 | Un-treated, mock 6hr |
| 3 | Un-treated, mock 9hr |
| 4 | Un-treated, mock 12hr |
| 5 | 10μM curcumin, mock 3hr |
| 6 | 10μM curcumin, mock 6hr |
| 7 | 10μM curcumin, mock 9hr |
| 8 | 10μM curcumin, mock 12hr |
| 9 | Un-treated, EV71 3hr |
| 10 | Un-treated, EV71 6hr |
| 11 | Un-treated, EV71 9hr |
| 12 | Un-treated, EV71 12hr |
| 13 | 10μM curcumin, EV71 3hr |
| 14 | 10μM curcumin, EV71 6hr |
| 15 | 10μM curcumin, EV71 9hr |
| 16 | 10μM curcumin, EV71 12hr |


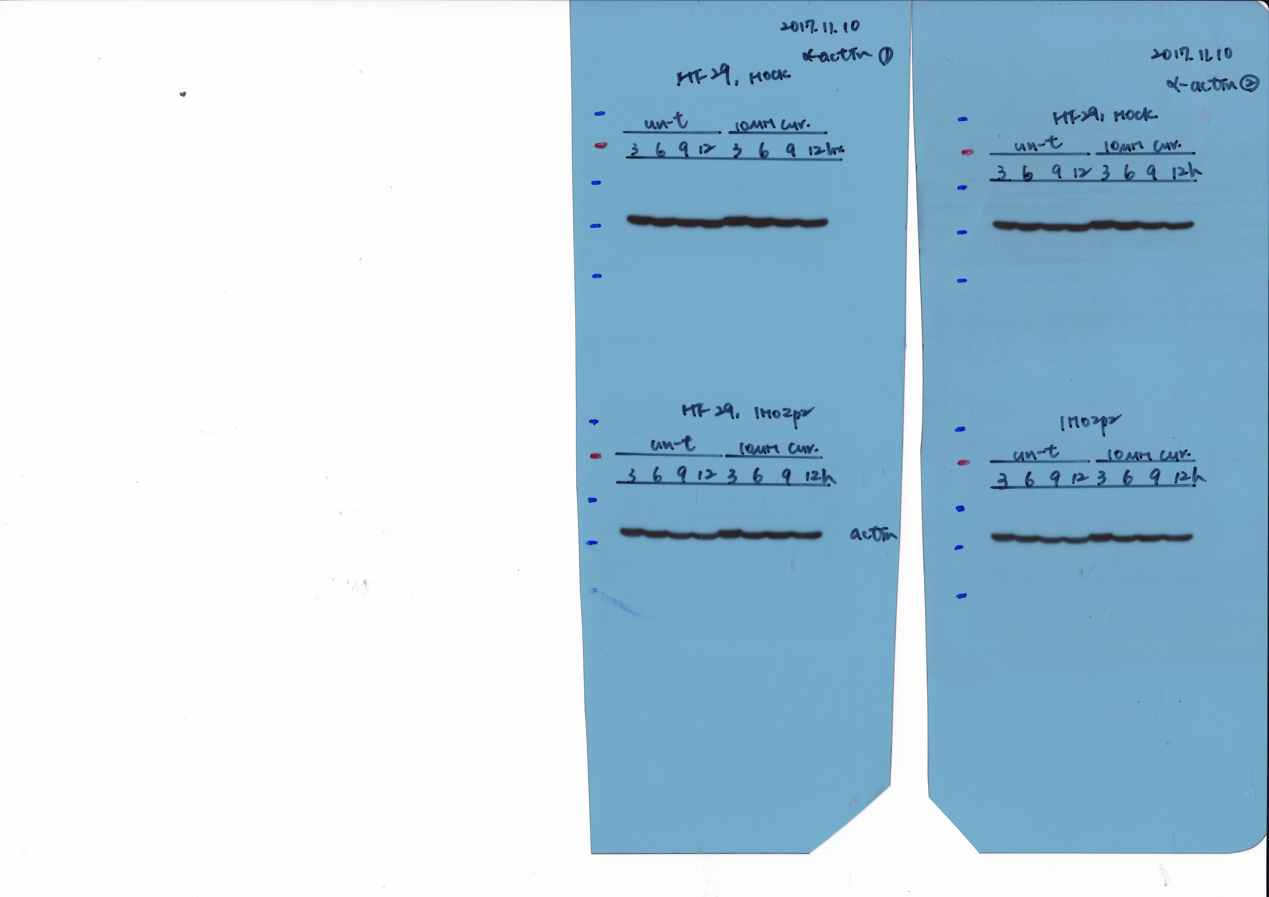

Supplement: S1 File — (ZIP) [file pone.0191617.s006.zip › Minimal manuscript dataset/S5 Fig.docx]
