# Supplementary material for: Inhibition of EV71 by curcumin in intestinal epithelial cells
Source: PLoS One. 2018 Jan 25;13(1):e0191617. doi: 10.1371/journal.pone.0191617 (PMC5784943; doi:10.1371/journal.pone.0191617)
Supplement: S1 File — (ZIP) [file pone.0191617.s006.zip › Minimal manuscript dataset/S4 Fig.docx]

**S4 Fig.** **Curcumin lacks of the virucidal activity.**

(A)


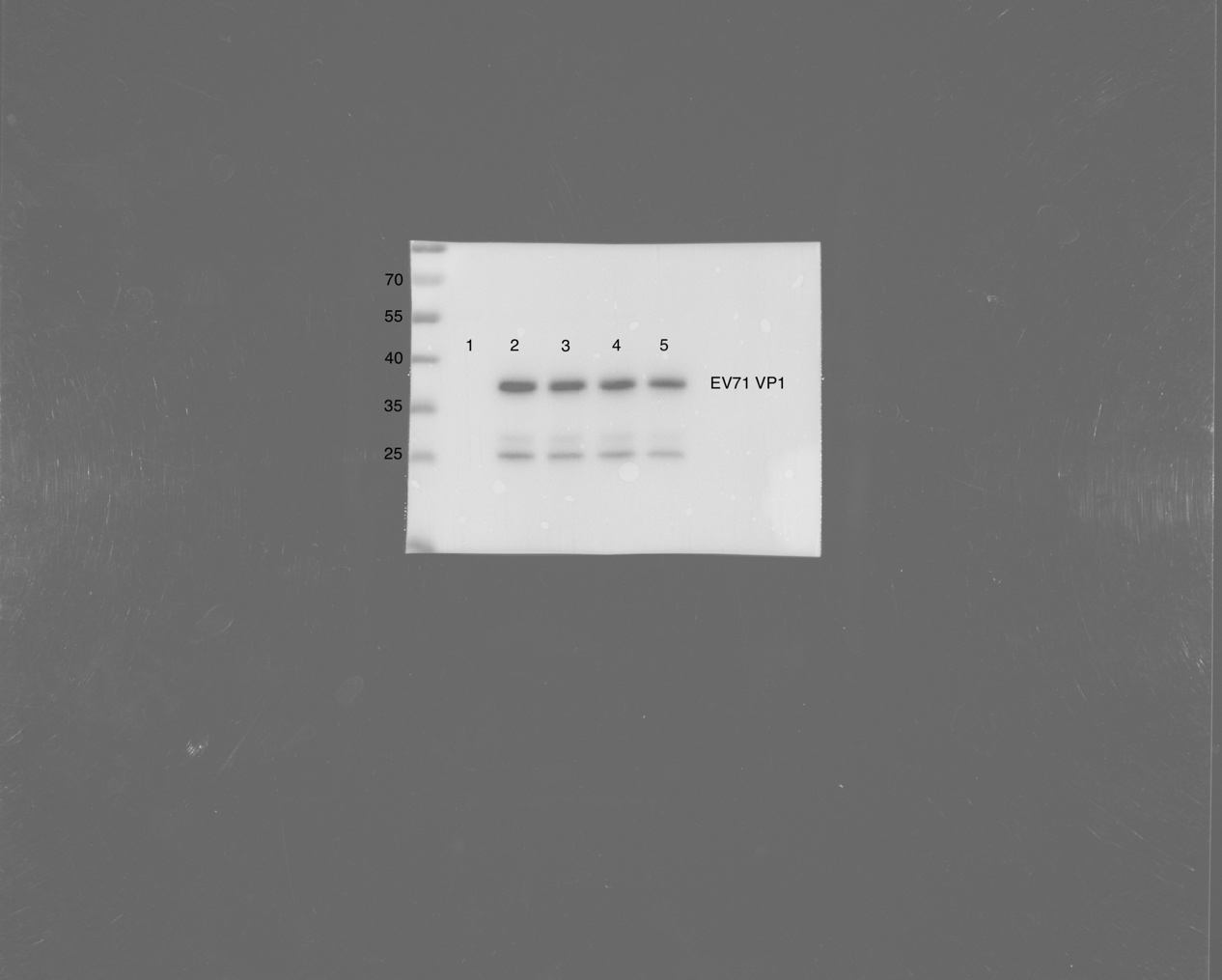

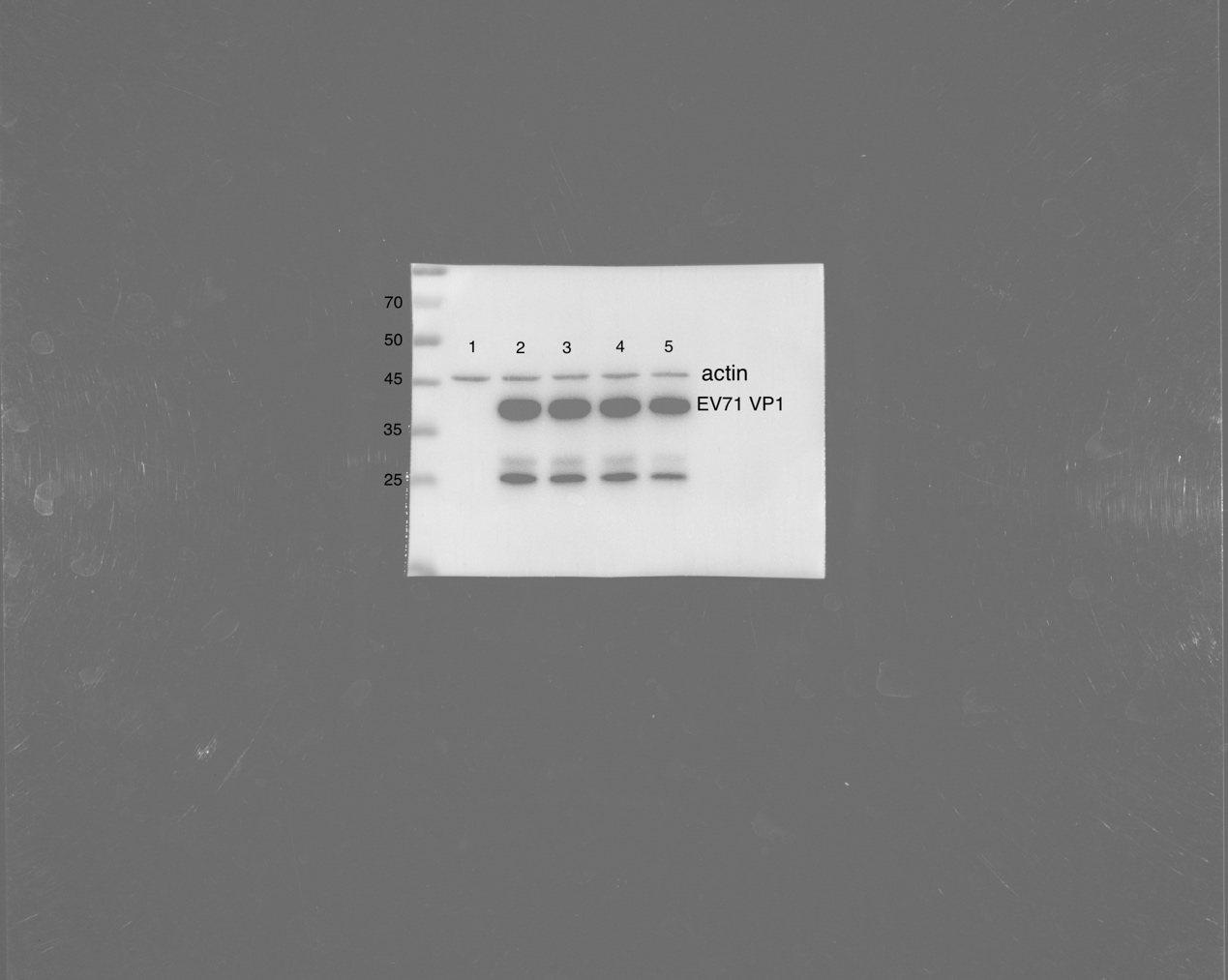


| Lane | sample |
| --- | --- |
| 1 | Mock |
| 2 | Un-treated+EV71 |
| 3 | 5μM curcumin+EV71 |
| 4 | 10μM curcumin+EV71 |
| 5 | 20μM curcumin+EV71 |
