# Supplementary material for: Inhibition of EV71 by curcumin in intestinal epithelial cells
Source: PLoS One. 2018 Jan 25;13(1):e0191617. doi: 10.1371/journal.pone.0191617 (PMC5784943; doi:10.1371/journal.pone.0191617)
Supplement: S1 File — (ZIP) [file pone.0191617.s006.zip › Minimal manuscript dataset/Fig 3.docx]

**Fig 3. Curcumin treatment increases the survival of host cells and suppresses EV71 replication.**

(A)

(B)

(C)


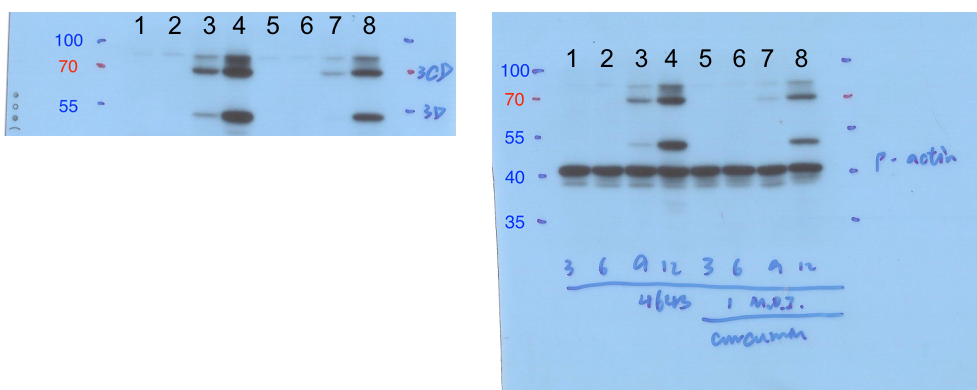


| Lane | Sample |
| --- | --- |
| 1 | Un-treated, EV71, 3hr |
| 2 | Un-treated, EV71, 6hr |
| 3 | Un-treated, EV71, 9hr |
| 4 | Un-treated, EV71, 12hr |
| 5 | 10μM curcumin, EV71, 3hr |
| 6 | 10μM curcumin, EV71, 6hr |
| 7 | 10μM curcumin, EV71, 9hr |
| 8 | 10μM curcumin, EV71, 12hr |

(D)
