# Supplementary material for: Inhibition of EV71 by curcumin in intestinal epithelial cells
Source: PLoS One. 2018 Jan 25;13(1):e0191617. doi: 10.1371/journal.pone.0191617 (PMC5784943; doi:10.1371/journal.pone.0191617)
Supplement: S1 File — (ZIP) [file pone.0191617.s006.zip › Minimal manuscript dataset/Fig 4.docx]

**Fig 4. Curcumin inhibits EV71 protein expression when added at the early stages of infection.**

(A)

| Lane | sample |
| --- | --- |
| 1 | mock |
| 2 | Un-treated+EV71 |
| 3 | Pre-treated 1hr+EV71 |
| 4 | Co-treated with EV71 |
| 5 | All treated+EV71 |
| 6 | Co-treated to 9hr p.i. +EV71 |
| 7 | Post 1hr~9hr +EV71 |
| 8 | Post 2hr~9hr+EV71 |
| 9 | Post 3hr~9hr+EV71 |


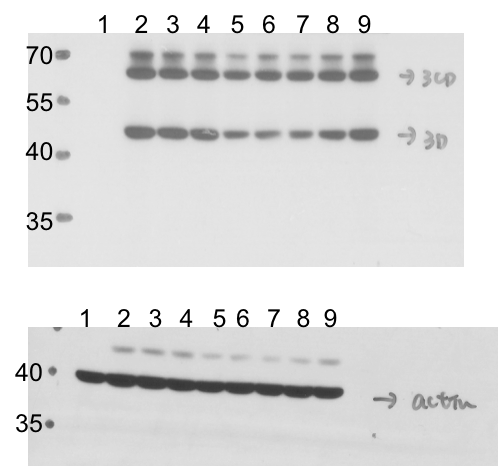


(B)

(C)

EV71 VP1

| Lane | sample |
| --- | --- |
| 1 | mock |
| 2 | Untreated+EV71 |
| 3 | 5μM cur.+EV71 |
| 4 | 10μM cur.+EV71 |
| 5 | 20μM cur.+EV71 |
| 6 | PR66+EV71 |


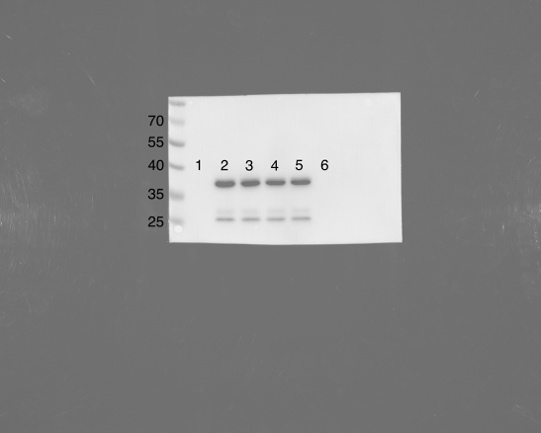


actin


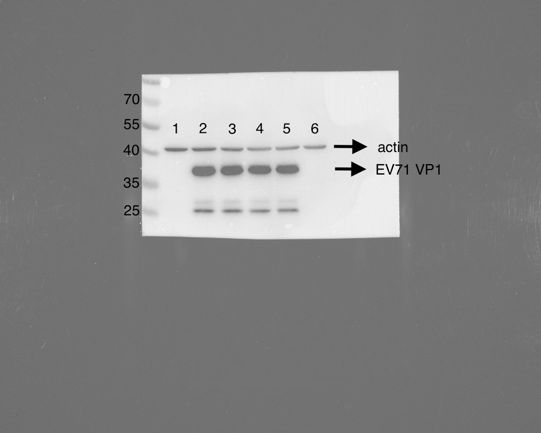


(D)
