# Supplementary material for: Inhibition of EV71 by curcumin in intestinal epithelial cells
Source: PLoS One. 2018 Jan 25;13(1):e0191617. doi: 10.1371/journal.pone.0191617 (PMC5784943; doi:10.1371/journal.pone.0191617)
Supplement: S1 File — (ZIP) [file pone.0191617.s006.zip › Minimal manuscript dataset/Fig 5.docx]

**Fig 5. Curcumin does not suppress EV71 by modifying MAPK pathways.**

(A)


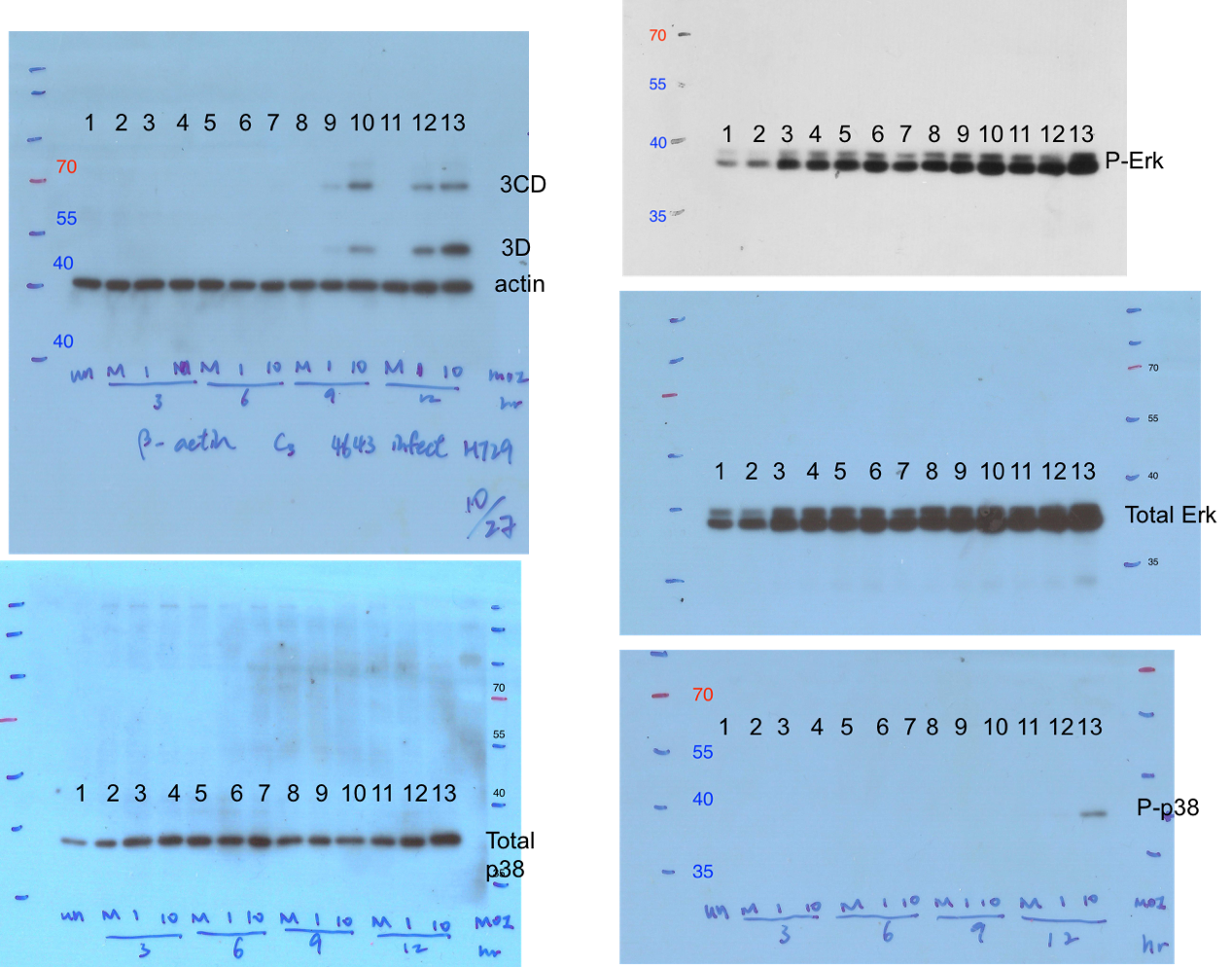


| Lane | sample |
| --- | --- |
| 1 | Un-treated and non-infection |
| 2 | Mock infection 3hr |
| 3 | 1MOI EV71 3hr |
| 4 | 10MOI EV71 3hr |
| 5 | Mock infection 6hr |
| 6 | 1MOI EV71 6hr |
| 7 | 10MOI EV71 6hr |
| 8 | Mock infection 3hr |
| 9 | 1MOI EV71 9hr |
| 10 | 10MOI EV71 9hr |
| 11 | Mock infection 12hr |
| 12 | 1MOI EV71 12hr |
| 13 | 10MOI EV71 12hr |

(B)


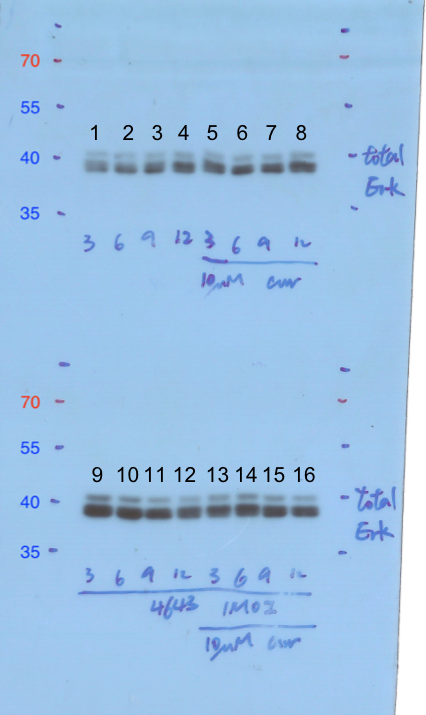

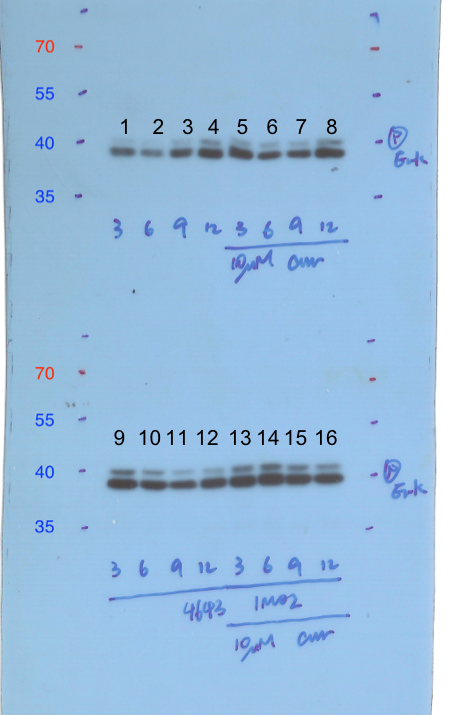


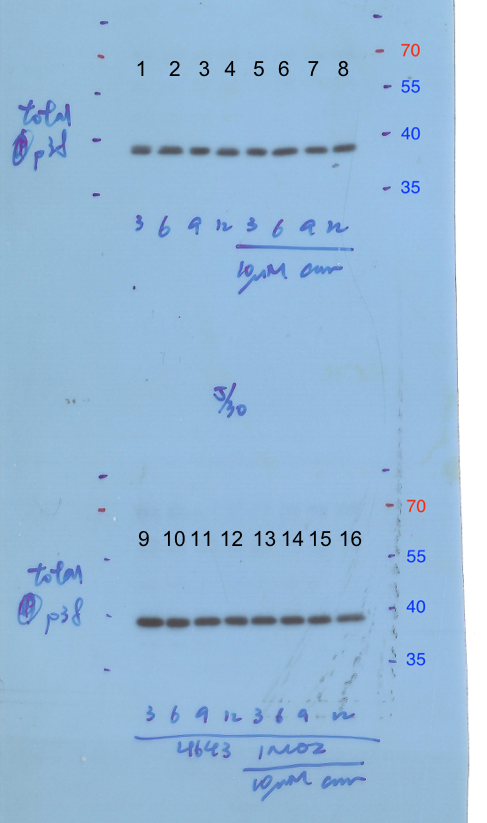

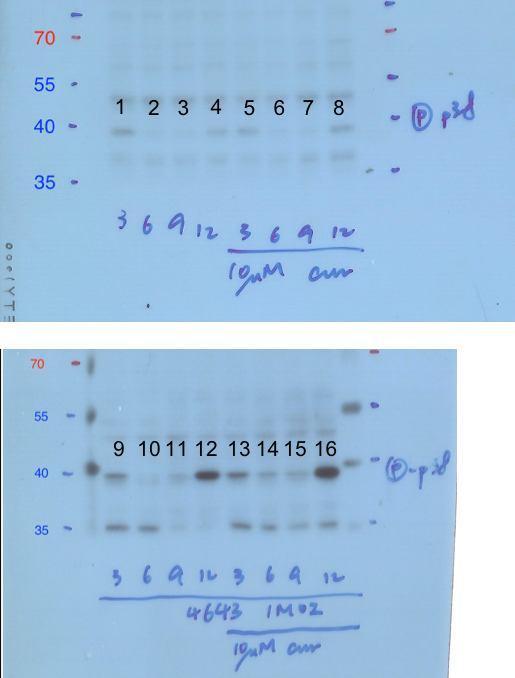


| Lane | sample |
| --- | --- |
| 1 | Mock 3hr un-treated |
| 2 | Mock 6hr un-treated |
| 3 | Mock 9hr un-treated |
| 4 | Mock 12hr un-treated |
| 5 | Mock 3hr 10μM curcumin |
| 6 | Mock 6hr10μM curcumin |
| 7 | Mock 9hr 10μM curcumin |
| 8 | Mock 12hr 10μM curcumin |
| 9 | 1MOI EV71 3hr un-treated |
| 10 | 1MOI EV71 6hr un-treated |
| 11 | 1MOI EV71 9hr un-treated |
| 12 | 1MOI EV71 12hr un-treated |
| 13 | 1MOI EV71 3hr 10μM curcumin |
| 14 | 1MOI EV71 6hr10μM curcumin |
| 15 | 1MOI EV71 9hr 10μM curcumin |
| 16 | 1MOI EV71 12hr 10μM curcumin |
